# ﻿DNA barcoding and morphology revealed the existence of seven new species of squat lobsters in the family Munididae (Decapoda, Galatheoidea) in the southwestern Pacific

**DOI:** 10.3897/zookeys.1188.114984

**Published:** 2024-01-03

**Authors:** Enrique Macpherson, Paula C. Rodríguez-Flores, Annie Machordom

**Affiliations:** 1 Centre d’Estudis Avançats de Blanes (CEAB-CSIC), C. acc. Cala Sant Francesc 14, 17300 Blanes, Girona, Spain Centre d’Estudis Avançats de Blanes Blanes Spain; 2 Department of Organismic and Evolutionary Biology, Museum of Comparative Zoology, Harvard University, 26 Oxford St., Cambridge MA 02138, USA Harvard University Cambridge United States of America; 3 Museo Nacional de Ciencias Naturales (MNCN-CSIC), José Gutiérrez Abascal, 2, 28006 Madrid, Spain Museo Nacional de Ciencias Naturales Madrid Spain

**Keywords:** Crustacea, integrative taxonomy, molecular characters, morphology, Pacific Ocean

## Abstract

Specimens of squat lobsters belonging to the family MunididaeAhyong et al., 2010, representing the genera *Garymunida* Macpherson & Baba, 2022, *Trapezionida* Macpherson & Baba, 2022 and *Typhlonida* Macpherson & Baba, 2022, were collected during several cruises around New Caledonia and Papua New Guinea, Southwest Pacific. The integrative study of these specimens revealed the presence of one new species in *Garymunida*, five in *Trapezionida* and one in *Typhlonida*. We describe and illustrate these new species, providing some new data on the taxonomy of several rare or scarcely studied species of *Trapezionida*. Molecular data from different markers (mitochondrial and nuclear) was also included, based on data availability, to support the taxonomic status of different species. Finally, a key to species for each genus is also provided.

## ﻿Introduction

The genera *Garymunida*, *Trapezionida*, and *Typhlonida* were described by Macpherson & Baba, in Machordom et al. (2022) in the recent revision of the family Munididae (Machordom et al. 2022). In this revision, the evolutionary history and systematics of the family are reconstructed using an integrative approach combining morphological and molecular analyses. After the analyses of more than 290 munidid species, these authors found some potentially new species, most of them morphologically related to well-known species, but genetically distinct. In the present study, our main objective is to describe these undescribed taxa using the material studied in the revision (Machordom et al. 2022), as well as additional specimens collected in recent expeditions carried out by the Muséum national d’Histoire naturelle of Paris, in the framework of the Tropical Deep-Sea Benthos program in Papua-New Guinea (expeditions Biopapua, Papua-niugini, Kavieng, Madeep), New Caledonia (Exbodi, Kanacono) and Chesterfield Islands (Ebisco, Kanadeep).

*Garymunida* Macpherson & Baba, in Machordom et al. 2022 is the sister genus of *Agononida* Baba & de Saint Laurent, 1996 and the species can be distinguished by the size of the distomesial process on the antennal article 1. At present, *Garymunida* includes 20 species, most of them distributed in the Indian and Pacific oceans, and two species present in the western Atlantic Ocean: *G.longipes* (A. Milne Edwards, 1880) and *G.schroederi* (Chace, 1939). *Trapezionida* Macpherson & Baba, in Machordom et al. 2022 is the most speciose genus in the family Munididae, containing more than 155 species, all from the Indian and Pacific Oceans. *Trapezionida* is the sister genus of *Gonionida* Macpherson & Baba, in Machordom et al. 2022, and they can be recognised by the shape of the thoracic sternite IV and the length of the P4 merus. The genus *Typhlonida* Macpherson & Baba, in Machordom et al. 2022 includes up to 25 species, all of them usually deep-water dwellers, occurring from the continental slope to the abyssal plain in the Atlantic, Indian, and Pacific oceans. The most distinctive character of most species in this genus is the small size of the cornea as well as the reduced size of some spines in the antennular and antennal peduncles (Machordom et al. 2022).

The existence of several new species of the family Munididae in New Caledonian and Papua-New Guinean waters suggests that, despite the enormous sampling effort in the area (Richer de Forges et al. 2013), the diversity of this group of decapods requires further study (De Grave et al. 2023). Recent studies in the Indian (e.g., Patel et al. 2022; Tiwari et al. 2022, 2023; Periasamy et al. 2023) and Pacific (e.g., Komai et al. 2023; Rodríguez-Flores et al. 2023) oceans confirm the numerous gaps that still exist in the taxonomy of squat lobsters. Therefore, in the present study, we describe and illustrate one new species of *Garymunida*, five of *Trapezionida*, and one of *Typhlonida*. We have also included new records and some remarks of a rare (*T.brachytes* (Macpherson, 1994)) and a complex of species (related to *T.leptitis* (Macpherson, 1994)) of *Trapezionida*. The dichotomous keys to species of the three genera have been included in order to facilitate future studies (see Suppl. material 1).

## ﻿Materials and methods

### ﻿Sampling and identification

The material (including the holotype of the new species) is located in the Muséum national d’Histoire naturelle, Paris (**MNHN**). The terminology and measurements follow Baba et al. (2009, 2011). The size of the specimens is indicated by the postorbital carapace length (CL), measured along the midline from the base of the rostrum to the posterior margin of the carapace. The rostrum was measured from its base (situated at the level of the orbit) to the distal tip. Measurements of appendages were taken in dorsal (pereopod 1), lateral (antennule, pereopods 2–4) and ventral (antenna) midlines. Abbreviations used are: **Mxp3**, maxilliped 3; **P1–P4**, pereopods 1–4; **M** = male; **F** = female; ovig. = ovigerous.

### ﻿Molecular data

The sequences of the different genes for each new species and comparative material were obtained from Machordom et al. (2022). The molecular data used for this study are deposited on GenBank (www.ncbi.nlm.nih.gov/genbank/) under the following accession numbers: *Garymunidanamora* sp. nov. (cytochrome oxidase subunit one COI: OP215605, 16S rRNA: OP195946), *Trapezionidabrachytes* (COI: OP215696, 16S: OP196034), *T.brevitas* sp. nov. (COI: OP215698, 16S: OP196036), *T.diluta* sp. nov. (COI: OP215699, 16S: OP196037), *T.leptitis* (16S: OP196069), *T.macilenta* sp. nov. (COI: OP215694, 16S: OP196032), *T.microtes* sp. nov. (COI: OP215712, 16S: OP196081), *T.pulex* sp. nov. (COI: OP215702 to 04, 16S: OP196040 to 42), *Typhlonidaeluminata* sp. nov. (COI: OP215758, 16S: OP196095, 18S rRNA -18S: OP196339).

Genetic distances between species were estimated using uncorrected divergences (p) calculated using PAUP version 4.0 (build 167) (Swofford 2004; see also Machordom et al. 2022).

## ﻿Systematic account

**Superfamily Galatheoidea Samouelle, 1819**

**Family Munididae Ahyong, Baba, Macpherson & Poore, 2010**

### ﻿Genus *Garymunida* Macpherson & Baba, 2022 in Machordom et al. 2022

#### ﻿ Garymunida namora sp. nov.

Taxon classificationAnimaliaDecapodaMunididae

7A15918B-92DB-5420-BEA6-D1FA3CF5B937

https://zoobank.org/4EA2A39A-F9A5-4DAC-AA42-C337942B452B

Figs 1 , 2

Garymunida sp. 2: Machordom et al. 2022: table 2, fig. 3.

##### Material.

***Holotype***: New Caledonia. Spanbios Stn CP5155, 5 July 2021, 20°05.9'S, 163°42.6'E, 573–575 m: male, 13.9 mm (MNHN-IU-2021-9330). ***Paratype***: Papua New Guinea. Biopapua Stn CP3682, 27 September 2010, 04°37.820'S, 149°27.940'E, 515–812 m: 1 female, 16.0 mm (MNHN-IU- 2011-3173).

##### Description.

***Carapace***: Approximately as long as wide, dorsally moderately convex. Transverse ridges usually microscopically granular, mostly interrupted, with dense, very short setae, and a few scattered long non-iridescent setae. Some granular scales on hepatic and anterior branchial regions. Main transverse striae on posterior part of carapace interrupted in cardiac region. Two strong epigastric, two protogastric behind epigastric and two parahepatic spines on gastric regions. One row of four or five strong branchiocardiac spines. Posterior margin with two median spines. Upper orbital margins slightly oblique; lower orbital margins visible dorsally, laterally with minute spine, mesially with low rounded process. Lateral margins moderately convex. Anterolateral spine strong, located at carapace anterolateral angle, reaching level of sinus between rostrum and supraocular spines. Second marginal spine before cervical groove well developed. Branchial margins feebly convex, with 4 spines. Rostrum spiniform, half as long as remaining carapace, slightly sinuous and nearly horizontal in lateral view. Supraocular spines exceeding midlength of rostrum and reaching or overreaching end of cornea, divergent, directed slightly upwards. Pterygostomian region unarmed ending anteriorly in rounded tip.

**Figure 1.**
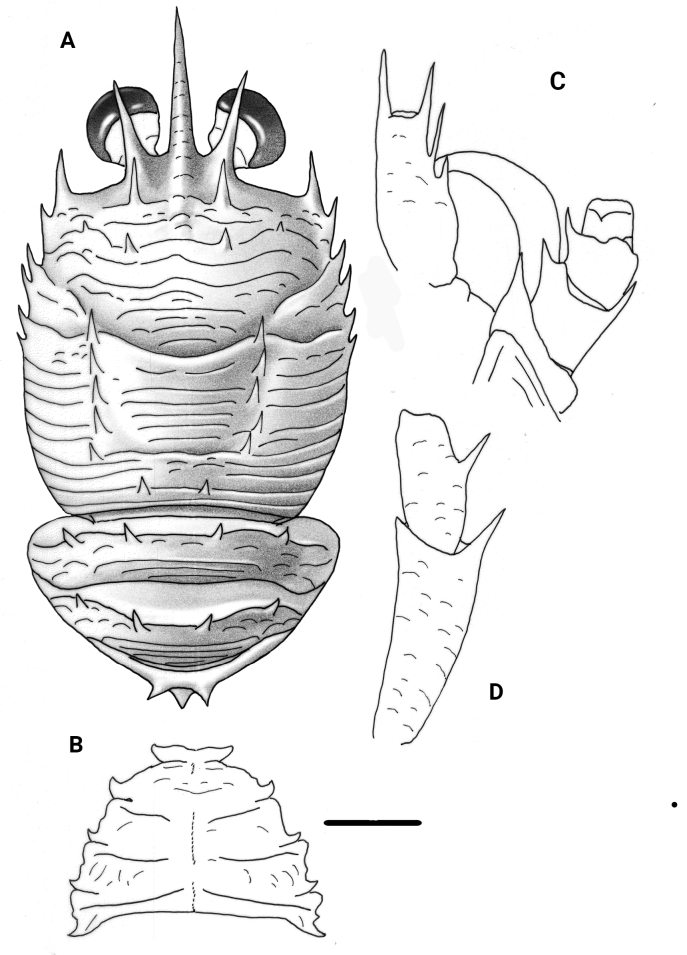
*Garymunidanamora* sp. nov., male holotype, 13.9 mm (MNHN-IU-2021-9330), New Caledonia **A** carapace and pleon, dorsal view **B** sternal plastron **C** cephalic region, showing antennular and antennal peduncles, ventral view **D** right Mxp3 ischium and merus, lateral view. Scale bars: 2.0 mm (**A, B**); 1.0 mm (**C, D**).

***Thoracic sternum***: 0.6× as wide as long. Sternite III with median shallow notch. Sternite IV with anterior part as wide than sternite III, with some short striae. Sternites IV–VI with a few striae on lateral sides. Sternite III ~ 5× as wide as long; sternite IV nearly 2.5× as wide as long, and 2× as wide as sternite III.

***Pleon***: Tergites II and III each with four spines on anterior margin; tergite IV with two median spines on anterior ridge and median spine on posterior ridge.

**Figure 2.**
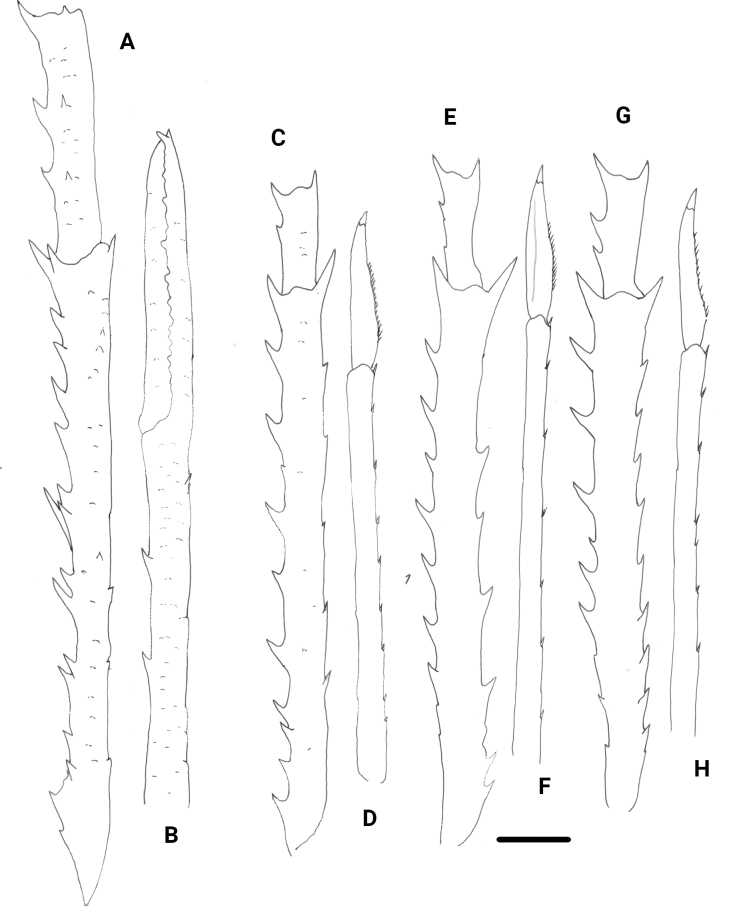
*Garymunidanamora* sp. nov., male holotype, 13.9 mm (MNHN-IU-2021-9330), New Caledonia **A** right P1, merus and carpus, dorsal view **B** right P1, palm and fingers, dorsal view **C** right P2, merus and carpus, lateral view **D** right P2, propodus and dactylus, lateral view **E** right P3, merus and carpus, lateral view **F** right P3, propodus and dactylus, lateral view **G** right P4, merus and carpus, lateral view **H** right P4, propodus and dactylus, lateral view. Scale bar: 10.0 mm.

***Eye***: Large, cornea dilated, maximum corneal diameter ~ 0.4 distance between bases of anterolateral spines.

***Antennule***: Article 1 (distal spines excluded), ~ 1/4 carapace length, slightly overreaching cornea, with two distal spines, mesial spine longer than lateral spine; two spines on lateral margin, proximal one small, located at midlength of segment, distal one long, not overreaching distolateral spine.

***Antenna***: Article 1 with one short process on mesial margin, reaching end of article 2; article 2 with two distal spines, mesial spine shorter than lateral spine, reaching midlength of article 3; article 3 with distomesial spine; article 4 unarmed.

***Mxp3***: Ischium ~ 2× length of merus measured along dorsal margin, distoventrally bearing strong spine. Merus with one strong median spine on flexor margin; extensor margin unarmed.

***P1***: 3.0× carapace length, with scattered long plumose setae. Merus with row of mesial spines; a few small, scattered spines on dorsal side. Carpus 5× as long as broad; with spines along mesial margin and a few minute spines on dorsal side. Palm 7.5× as long as broad, with a few small dorsal spines; one or two spines along mesial and lateral margins. Fingers 0.8× palm length.

***P2–P4***: Long and slender, with numerous long non-plumose and non-iridescent setae along extensor margin of articles. P2 3.0× carapace length. Meri slightly shorter posteriorly (P2 merus as long as P3 merus; P3 merus 0.9 length of P4 merus); P2 merus 1.5× carapace length, 13.0–13.5× as long as broad, 1.4× longer than P2 propodus; P3 merus 12.5× as long as broad; P4 merus 11.5× as long as broad. Extensor and flexor margins of P2–P4 meri with row of 9–11 and 6–10 spines, respectively, proximally diminishing spines; lateral sides unarmed, more squamate in P4. P2–P4 carpi with 2–4 spines on extensor margin; lateral surface with several granules sub-paralleling extensor margin; flexor margin with distal spine. Propodi 14.5–17.0× as long as broad; extensor margin unarmed; flexor margin with 7–9 slender movable spines, distal end without fixed spine. Dactyli slender, length 0.4 that of propodi; flexor margin with 13–17 movable spinules, proximal and distal fourth unarmed, without a spinule at the base of the unguis; P2 dactylus 8.2× as long as wide.

##### Genetic data.

COI, 16S.

##### Etymology.

"*Namora*" is a mythological woman participating in the creation of New Guinea. Used as noun in apposition.

##### Remarks.

The closest species to the new species is *G.procera* (Ahyong & Poore, 2004), from New Caledonia, Queensland, New South Wales and New Zealand, *G.imitata* (Macpherson, 2006), from French Polynesia, and *G.soelae* (Baba, 1986), from Kyushu-Palau Ridge, Taiwan, Indonesia, SW Australia, and Fiji. These species have the pleomere IV tergite armed with median spine on posterior transverse ridge, the article 1 of the antennal peduncle with moderate-sized process, not reaching article 4, and a pair of protogastric spines behind median pair of epigastric spines. However, the new species differ easily from the other three species in the following characters:

- The cardiac region is unarmed in the new species, whereas there are some median spines in the other three species.
- The posterior ridge of the carapace has two median spines in *G.namora*, whereas there are six spines in the other three species.
- The divergences between *G.namora* and the morphologically or phylogenetically closest species are ~ 14% for COI (respect to *G.imitata*, *G.procera* or *G.longipes*). These values drop to 6.1–8.6% when 16S is analysed and comparisons are made respect to *G.simillima*, *G.imitata*, *G.laurentae*, *G.procera*, or *G.longipes*.

##### Distribution.

Papua-New Guinea and New Caledonia, between 515 and 812 m.

### ﻿Genus *Trapezionida* Macpherson & Baba, 2022 in Machordom et al. 2022

#### ﻿ Trapezionida brachytes

Taxon classificationAnimaliaDecapodaMunididae

(Macpherson, 1994)

7A3104AF-AFBF-50AF-970D-55A6CE38E641

Fig. 3

Munida brachytes Macpherson 1994: 450, fig. 8. — Baba 2005: 260. — Baba et al. 2008: 89.
Trapezionida aff. apheles : Machordom et al. 2022: table 2, suppl. figs S1–S6.

##### Material.

***Holotype***: New Caledonia, Smib 5 Stn 86, 13 September 1989, 22°19.8´S, 168°42.8´E, 320 m: male, 3.7 mm (MNHN Ga 2580).

New Caledonia, Chesterfield Islands. Ebisco Stn CP2495, 6 October 2005, 24°44.11'S, 159°42.9'E, 217–350 m: 1 male, 4.4 mm (MNHN- IU-2016-5803). — Stn DW2526, 9 October 2005, 22°47.492'S, 159°22.890'E, 340–355 m: 1 male, 4.4 mm (MNHN-IU-2013-19882). Kanadeep 1 Stn DW5006, 19 September 2017, 22°07'S, 159°19'E, 340–550 m: 1 ovigerous female, 4.5 mm, 1 female, 4.4 mm (MNHN-IU-2017-2519). — Stn DW5011, 19 September 2017, 22°13'S, 159°03'E, 320–350 m: 1 male, 5.2 mm (MNHN-IU-2014-13918), 1 female, 3.7 mm (MNHN-IU-2017-2926).

**Figure 3.**
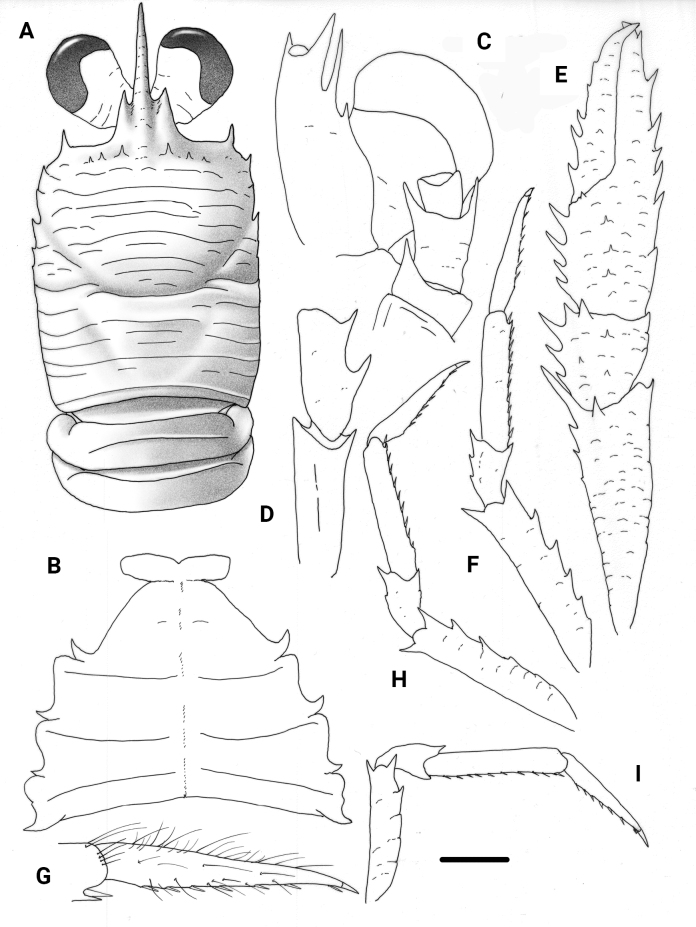
*Trapezionidabrachytes* (Macpherson, 1994), female, 3.7 mm (MNHN-IU-2017-2926), New Caledonia **A** carapace and pleon, dorsal view **B** sternal plastron **C** cephalic region, showing antennular and antennal peduncles, ventral view **D** right Mxp3 ischium and merus, lateral view **E** right P1, dorsal view **F** right P2, lateral view **G** dactylus of right P2, lateral view **H** right P3, lateral view **I** right P4, lateral view. Scale bars: 1.0 mm (**A, E, F, H, I**); 0.5 mm (**B, C, D, G**).

##### Diagnosis.

(modified from Macpherson 1994) Carapace slightly longer than broad, with a few secondary striae between main transverse ridges. Gastric region with three or four pairs of epigastric spines. Parahepatic, branchial dorsal and postcervical spines absent. Frontal margins transverse. Lateral margins slightly convex. First lateral spine slightly mesial to anterolateral angle, short, clearly not reaching level of sinus between rostrum and supraocular spines. Branchial margins with five minute spines, posterior spines sometimes obsolescent. Rostrum spiniform. Supraocular spines short, not reaching midlength of rostrum and clearly not reaching end of cornea. Surface of thoracic sternum smooth; sternite IV trapezoidal, anterior margin contiguous to sternite III along ¾ of its length. Pleomere tergites unarmed, tergites II and III each with one transverse ridge on tergite behind anterior ridge. Cornea much wider than peduncle. Antennular article 1 with two well-developed distal spines, distomesial spine as long as or shorter than distolateral. Antennal article 1 with short distomesial spine nearly reaching midlength of article 2; article 2 with distomesial and distolateral spines reaching or nearly reaching end of article 3. Extensor margin of Mxp3 merus unarmed. P1 palm with well-developed spines along lateral and mesial margins continuing along fixed and movable fingers, respectively. Extensor margins of P2–P4 meri unarmed, one distal spine only; flexor margins with some well-developed spines followed proximally by several eminences; P2–P4 dactyli slender, as long as or slightly shorter than propodi; with movable spinules along nearly entire flexor margin, with ultimate spinule near unguis (sometimes lost, as in holotype); P2 dactylus 5.5–6.3× as long as wide; P4 merus > ½ length of P2 merus.

##### Genetic data.

COI, 16S.

##### Remarks.

*Trapezionidabrachytes* was only known by one male collected in New Caledonia. The new material collected in New Caledonia and Chesterfield Islands agree quite well with the holotype, although the distomesial spine of the antennular article 1 is shorter than the distolateral spine (subequal in the holotype). Furthermore, the distalmost movable spinule along the flexor margin of the P2–P4 dactyli can be lost (although the insertion point is always present). Therefore, these characters should be considered with caution because these spines/spinules can be broken or regenerating in some specimens.

Morphologically and genetically the closest species of *T.brachytes* is *T.stia* (Macpherson, 1994), also known from New Caledonia and Chesterfield islands.

*Trapezionidastia* and *T.brachytes* can be distinguished by the following characters:

- Pleomeres III and IV tergites have an additional ridge behind the anterior ridge in *T.stia*, whereas these additional ridges are absent in the new species.
- P2–P4 dactyli are shorter and stouter in *T.stia* than in *T.brachytes*. The dactyli are ~ 2/3 the propodi length in *T.stia*, whereas they are as long as or slightly shorter than propodi in *T.brachytes*. The P2 dactylus is 6.1–6.3× as long as wide in the new species, instead of 4× in *T.stia*. Finally, the terminal 1/3 of the flexor margin of the dactyli are unarmed in *T.stia*, whereas there are spines along the entire margin in *T.brachytes*.
- Genetically *T.brachytes* is different from *T.stia* (5.96% 16S) and (10.6% COI).

##### Distribution.

New Caledonia and Chesterfield Islands, between 310 and 550 m.

#### ﻿ Trapezionida brevitas sp. nov.

Taxon classificationAnimaliaDecapodaMunididae

F3CDEC18-66D3-5C06-AB0C-8CB9CC214F7E

https://zoobank.org/BB8E911E-4EC6-438C-8D86-81D6DD43BCE2

Fig. 4

Trapezionida aff. fornacis : Machordom et al. 2022: table 2, suppl. figs S1–S6.

##### Material.

***Holotype***: New Caledonia. Smib 8 Stn DW163, 28 January 1993, 24°49.12'S, 168°08.93'E, 310–460 m: male, 4.1 mm (MNHN-IU-2017-1336). ***Paratype***: New Caledonia. Norfolk 2 Stn DW2024, 21 October 2003, 23°27.92'S, 167°50.90'E, 370–371 m: 1 male, 4.6 mm (MNHN-IU-2014-13973).

##### Description.

***Carapace***: Slightly longer than broad, moderately convex, with a few secondary striae and scales between main transverse ridges. Dorsal ridges with dense short plumose setae and a few scattered long iridescent setae. Gastric region with four pairs of epigastric spines, longest pair behind supraocular spines, one pair between largest pair of spines. One parahepatic, two branchial dorsal and one postcervical spine on each side. Frontal margins oblique. Lateral margins slightly convex. First lateral spine at anterolateral angle, moderately long, clearly not reaching level of sinus between rostrum and supraocular spines; one–two small spines in front of anterior branch of cervical groove; end of anterior branch of cervical groove without tuft of iridescent setae. Branchial margins slightly convex, with four spines. Rostrum spiniform, ~ 0.6× length of remaining carapace, slightly upwards directed, dorsally slightly carinated. Supraocular spines reaching midlength of rostrum and not reaching end of cornea, slightly divergent, directed slightly upwards. Pterygostomian region unarmed, ending in blunt angle.

***Thoracic sternum***: 0.8× as long as broad. Surface of thoracic sternites IV–VI smooth. Sternite IV trapezoidal, anterior margin contiguous to sternite III along ¾ of its length. Sternite III 3.5× as wide as long; sternite IV 2.5× as wide as long, and 2.3× as wide as sternite III.

***Pleon***: Anterior ridge of pleomere tergites unarmed; tergites II and III each with one uninterrupted transverse ridge on tergite behind anterior ridge, absent on tergites IV and V; some iridescent setae on each side of anterior ridges of tergites; posteromedian margin of tergite VI straight.

***Eye***: Ocular peduncle as long as broad. Cornea dilated, maximum corneal diameter 0.4 distance between bases of anterolateral spines.

***Antennule***: Article 1 (distal spines excluded) ~ 0.3× carapace length, 2× as long as wide (excluding spines), slightly exceeding end of cornea, with two distal spines, distomesial subequal or slightly longer than distolateral; two spines on lateral margin, proximal one short, located at midlength of segment, distal one long, nearly reaching end of distolateral spine.

***Antenna***: Article 1 with distomesial spine slightly exceeding distal margin of article 2. Article 2 with subequal distomesial and distolateral spines, nearly reaching end of article 3. Article 3 unarmed.

***Mxp3***: Ischium with strong distal spine on flexor margin. Merus shorter than ischium; flexor margin with 2 spines, proximal stronger than distal; extensor margin with small distal spine. Carpus unarmed.

***P1***: 3.5–3.7× carapace length, with minute scales, short plumose setae on each scale, and some scattered long spines. Merus 1.3× length of carapace, 2.2× as long as carpus, with some dorsal and mesial spines, distomesial spine strong, not reaching first quarter of carpus. Carpus 0.8 length of palm, 2.3× as long as broad, with some spines along mesial and dorsal sides. Palm 1.8–2.3× as long as broad, with some small dorsal spines; row of spines along mesial and lateral margins. Fingers as long as palm; movable with small proximal spine at base, otherwise unarmed, with minute distal spine on fixed finger.

***P2–P4***: Moderately long and slender, covered with setose scales, with some long plumose setae and some long iridescent setae along extensor margin of articles. P2 2.0× carapace length. Meri shorter posteriorly (P3 merus 0.9× length of P2 merus, P4 merus 0.8× length of P3 merus); P2 merus 0.8× length of carapace, 4.2× as long as broad, 1.4× as long as P2 propodus; P3 merus 4.5×as long as broad, 1.3× length of P3 propodus; P4 merus 3.5× as long as broad, 1.1× length of P4 propodus. Extensor margins of P2–P3 meri with row of seven–eight proximally diminishing spines, one small distal spine on P4; flexor margins distally with one–two spines followed proximally by several eminences; lateral sides unarmed. Carpi with three spines on extensor margin of P2–P3, one minute distal spine on P4; lateral surface with several granules sub-paralleling extensor margin on P2–P4; flexor margin with distal spine. Propodi 4.5–5.5× as long as broad; extensor margin unarmed; flexor margin with four–ten slender movable spines on P2–P4, distal end with one minute fixed spine. Dactyli slender, length 0.6 that of propodi; flexor margin with six–seven movable spinules, without spinule at base of unguis, distal third unarmed; P2 dactylus 4.0× as long as wide. P4 merocarpal articulation reaching anterior end of cervical groove; P4 merus > ½ length of P2 merus.

**Figure 4.**
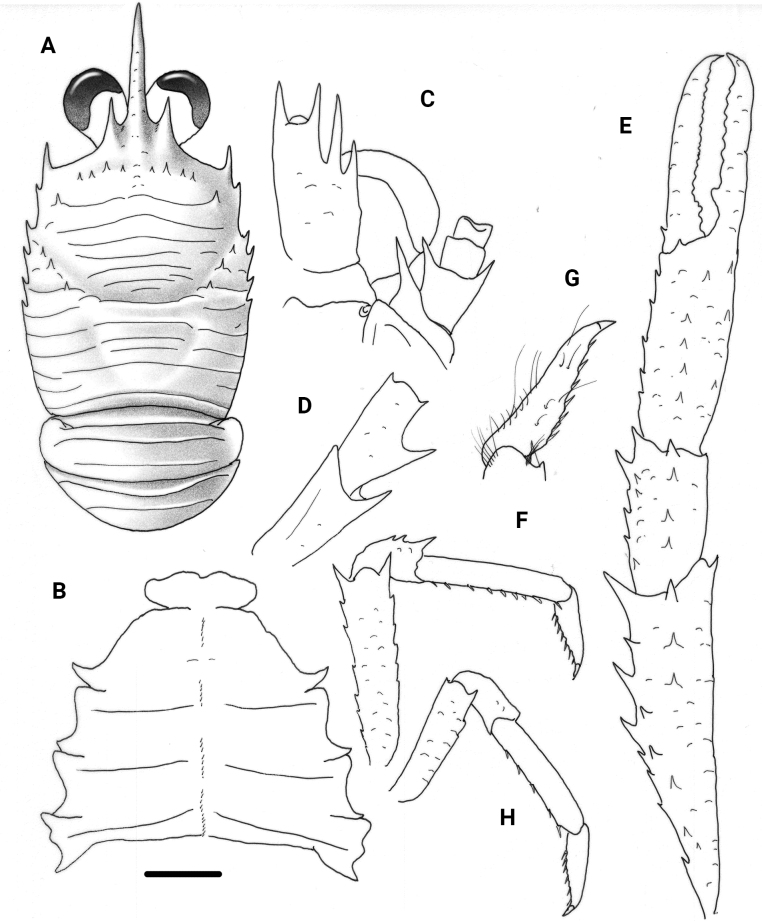
*Trapezionidabrevitas* sp. nov., male holotype, 4.1 mm (MNHN-IU-2017-1336), New Caledonia **A** carapace and pleon, dorsal view **B** sternal plastron **C** cephalic region, showing antennular and antennal peduncles, ventral view **D** right Mxp3 ischium and merus, lateral view **E** right P1, dorsal view **F** right P2, lateral view **G** dactylus of right P2, lateral view **H** right P4, lateral view. Scale bars: 1.0 mm (**A, E, F, H**); 0.5 mm (**B, C, D, G**).

##### Genetic data.

COI, 16S.

##### Etymology.

From the Latin, *brevitas*, shortness, in reference to the small size of the species.

##### Remarks.

*Trapezionidabrevitas* belongs to the group of species having four spines on the branchial lateral margins of the carapace, frontal margins oblique, thoracic sternites without granules or carinae, anterior ridge of the pleomeres II and III tergites unarmed, article 1 of antennule with subequal distal spines or distomesial spine slightly longer than distolateral, extensor margin of Mxp3 merus with small distal spine and flexor margin of P2–P4 dactyli with spinules along entire margin. The new species is closely related to *T.fornacis* (Macpherson, 2006) and *T.descensa* (Macpherson, 2006), both from French Polynesia. However, the three species differ from each other in some characters.

The differences between *T.brevitas* and *T.fornacis* are as follows:

- The dorsal carapace surface has two branchial dorsal spines in *T.brevitas*, and only one in *T.fornacis*.
- The pleomeres II and III tergites each with one uninterrupted transverse ridge on tergite behind anterior ridge in the new species, these ridges are interrupted or scale-like in *T.fornacis*.
- The distal spines of the antennular article 1 are subequal or slightly different in the new species, whereas the distomesial spine is clearly longer than the distolateral in *T.fornacis*.
- The distomesial spine of the antennal article 1 is very long, reaching the end of the antennal peduncle in *T.fornacis*, whereas this spine only slightly exceeds the antennal article 2.
- The dorsal surface of P1 palm is unarmed in *T.fornacis*, whereas it has numerous spines in *T.brevitas*.

Genetically both species are different. *T.brevitas* showed high divergence values with *T.fornacis* (15.9% COI).

The differences between *T.brevitas* and *T.descensa* are as follows:

- The dorsal carapace surface has two branchial dorsal spines in *T.brevitas*, and only one in *T.descensa*.
- P2–P4 are more slender in *T.descensa* (P2 merus and dactylus, 6 and 5× as long as wide, respectively) than in *T.brevitas* (P2 merus and dactylus, 4 and 4× as long as wide, respectively).
- Genetically both species are different. *T.brevitas* showed divergence values with *T.descensa* of ~ 4.3% for COI).

##### Distribution.

New Caledonia, at 310–460 m.

#### ﻿ Trapezionida diluta sp. nov.

Taxon classificationAnimaliaDecapodaMunididae

F472CA3E-2659-5AE0-A1FE-B6536ED39BCD

https://zoobank.org/DF07441C-1C86-49E7-9BEB-BDDF8141E36A

Fig. 5

Trapezionida aff. gordoae : Machordom et al. 2022: table 2, suppl. figs S1–S6.

##### Material.

***Holotype***: New Caledonia. Lifou 2000 Stn DW1462, 9 November 2000, 20°47.1'S, 167°03.2'E, 70–120 m: male, 4.3 mm (MNHN-IU-2016-9652). ***Paratypes***: New Caledonia. Lifou 2000 Stn DW1462, 9 November 2000, 20°47.1'S, 167°03.2'E, 70–120 m: 3 males, 3.5–4.6 mm, 1 ovigerous female, 3.9 mm (MNHN-IU-2014-13974).

##### Description.

***Carapace***: 1.2× as long as broad, moderately convex, with a few secondary striae between main transverse ridges. Ridges with short non-iridescent setae and few scattered long iridescent setae. Intestinal region without scales. Gastric region with four or five pairs of epigastric spines, longest pair behind supraocular spines, one pair between longest pair; one median protogastric spine. One parahepatic, one anterior branchial, and one postcervical spine on each side. Frontal margins oblique. Lateral margins slightly convex and convergent posteriorly. First lateral spine at anterolateral angle, long, not reaching level of sinus between rostrum and supraocular spines; second spine well developed, in front of anterior branch of cervical groove, < ½ length of first spine. Branchial margins straight, with three or four spines. Rostrum spiniform horizontal, ~ 0.4–0.6× length of remaining carapace, not dorsally carinated. Supraocular spines reaching midlength of rostrum and not reaching end of cornea, subparallel, slightly upwards directed. Pterygostomian flap unarmed, ending in round tip.

**Figure 5.**
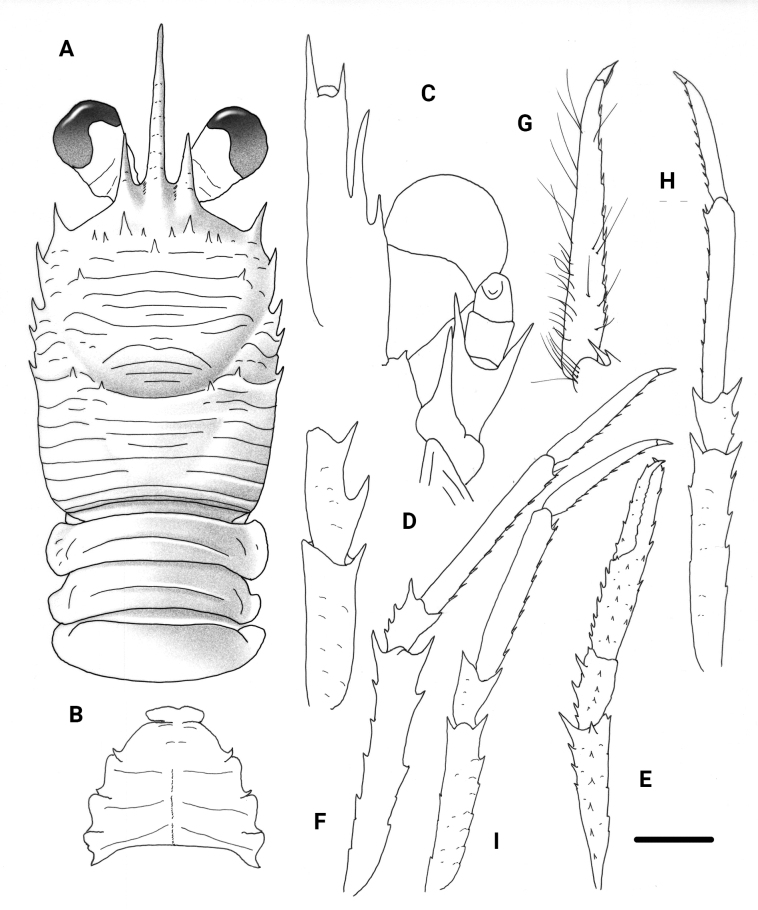
*Trapezionidadiluta* sp. nov., male holotype, 4.3 mm (MNHN-IU-2016-9652), New Caledonia **A** carapace and pleon, dorsal view **B** sternal plastron **C** cephalic region, showing antennular and antennal peduncles, ventral view **D** right Mxp3 ischium and merus, lateral view **E** right P1, dorsal view **F** right P2, lateral view **G** dactylus of right P2, lateral view **H** left P3, lateral view **I** right P4, lateral view. Scale bars: 1.0 mm (**A, B, F, H, I**); 0.5 mm (**C, D, G**); 2.0 mm (**E**).

***Thoracic sternum***: 0.8× as long as wide. Surface of thoracic sternites IV–VI smooth, only a few short scales on sternite IV. Sternite III 3.5× as wide as long. Sternite IV trapezoidal, anterior margin contiguous to sternite III along ¾ of its length; 2.5× as wide as long, and 2.0× as wide as sternite III.

***Pleon***: Ridges of pleomeres unarmed; tergites II and III each with uninterrupted transverse ridge on tergite behind anterior ridge: tergites IV and V with anterior ridge only; posteromedian margin of tergite VI straight.

***Eye***: Ocular peduncle longer than broad. Cornea dilated; maximum diameter 0.4× distance between bases of anterolateral spines.

***Antennule***: Article 1 (distal spines excluded) very long, ~ 0.5× carapace length, 3.5× as long as wide (excluding spines), clearly overreaching end of cornea, with two distal spines, mesial spine longer than lateral; two spines on lateral margin, proximal one short, located at midlength of segment, distal one long, not reaching end of distal spines.

***Antenna***: Article 1 with distomesial spine barely exceeding article 2. Article 2 with distomesial spine, exceeding article 3; distolateral spine as long as or slightly larger than distomesial, exceeding article 3. Articles 3 and 4 unarmed.

***Mxp3***: Ischium with well-developed spine on flexor distal margin. Merus shorter than ischium; flexor margin with two spines, median slightly stronger than distal spine; extensor margin unarmed. Carpus unarmed.

***P1***: 2.3–3.0× carapace length, with scattered long plumose setae, and some long iridescent setae; some short setae on spines and scales. Merus 1.0–1.1 length of carapace, 2.0–2.2× as long as carpus, with some dorsal and mesial spines; distal spines strong, distomesial spine barely reaching proximal fourth of carpus. Carpus 0.8–1.0 length of palm, 2.3–2.5× as long as broad; with spines along mesial and dorsal sides. Palm 2.3–2.8× as long as broad, with row of small dorsal spines; one row of spines along mesial and lateral margins, continuing along movable and lateral fingers, respectively. Length of fingers 1.1–1.3× that of palm.

***P2–P4***: Long and slender, with some short setae and some scattered iridescent setae along extensor margins of all articles. P2 2.1–2.2× carapace length. Meri shorter posteriorly (P3 merus 0.8–0.9× length of P2 merus, P4 merus 0.8–0.9× length of P3 merus); P2 merus 0.7–0.8× carapace length, 5.8–6.5× as long as broad, 1.2–1.3× as long as P2 propodus; P3 merus 6.0–6.2× as long as broad, 1.2–1.2× as long as P3 propodus; P4 merus 4.5–4.7× as long as broad, as long as P4 propodus. Extensor margins of meri with row of 5–7 proximally diminishing spines on P2–P3, one or two spines on P4; flexor margins with three or four spines followed proximally by several eminences; lateral sides unarmed. Carpi with three or four spines on extensor margin of P2–P3, one distal on P4; lateral surface with several granules sub-paralleling extensor margin on P2–4; flexor margin with well-developed distal spine. Propodi 6.5–7.6× as long as broad; extensor margin unarmed; flexor margin with eight–ten slender movable spines on P2–P4, one fixed distal spine. Dactyli slender, length 0.7–0.8× that of propodi; flexor margin with 10–11 movable spinules along entire border, with ultimate spinule at base of unguis, penultimate spine equidistant between antepenultimate and ultimate spines; P2 dactylus 7.4–7.5× as long as wide. P4 merocarpal articulation exceeding anterior end of cervical groove; P4 merus > ½ length of P2 merus.

##### Genetic data.

COI, 16S.

##### Etymology.

From the Latin, *dilutus*, thin, in reference to the shape of the antennular peduncle.

##### Remarks.

*Trapezionidadiluta* belongs to the group of species having the anterior ridge of the pleomere II tergite unarmed, the thoracic sternites smooth and the antennular article 1 very slender, exceeding eye, and with the distomesial spine longer than the distolateral spine. The new species is close to *T.macilenta* sp. nov., from Papua-New Guinea (see below under the Remarks of this species).

##### Distribution.

New Caledonia, 70–120 m.

#### ﻿ Trapezionida leptitis

Taxon classificationAnimaliaDecapodaMunididae

(Macpherson, 1994)

FA06AC05-CA6A-5B17-8438-CC4163ACAC29

Munida leptitis Macpherson 1994: 487, fig. 27. — Baba 2005: 267 (in part). — Macpherson 2006: 318 (in part). — Baba et al. 2008: 104 (in part). — Baba et al. 2009: 171, figs 151–152. — Macpherson 2013: 301 (in part).
Trapezionida leptitis : Machordom et al. 2022: table 2, suppl. figs S1–S6.

##### Material.

***Holotype***: New Caledonia, Loyalty Islands, Musorstom 6 Stn DW431, 18 February 1989, 20°22.25'S, 166°10'E, 21 m: female, 3.4 mm (MNHN-IU-2014-10854 (= MNHN Ga2810). ***Paratype***: New Caledonia, Loyalty Islands, Musorstom 6 Stn DW392, 13 February 1989, 20°47.32'S, 167°04.60'E, 340 m: 1 ovigerous female, 4.4 mm (MNHN-IU-2017-8971). French Polynesia, Tarasoc Stn DW3413, 13 October 2009, 16°34'S, 151°46'W, 385–486 m: 2 males, 4.5–6.0 mm, 4 ovigerous females, 4.0–5.2 mm, 1 female, broken (MNHN-IU-2010-5816), 1 ovigerous female, 5.3 mm (MNHN-IU-2016-520). — Lifou 2000 Stn 1650, 15/18 November 2000, 20°54.15'S, 167°01.7'E, 120–250 m: 2 female, 2.5–3.4 mm (MNHN-IU-2016-519). — Stn 1442, 13–14 November 2000, 20°46.4'S, 167°02'E, 47 m: 1 ovigerous female, 4.2 mm (MNHN-IU-2014-13820).

##### Colour in life

**(from Baba et al. 2009).** Ground colour of body and appendages orange. P1 with numerous red spots, mesial borders of articles darker than lateral margins; proximal half of fingers whitish, distal half reddish, tips white. P2–P4 scattered with red spots; dactyli whitish.

##### Genetic data.

COI, 16S.

##### Remarks.

This species has been cited in numerous localities from the West and Central Pacific. However, as it was mentioned in previous papers (see above) some slight morphological differences among specimens from different areas were observed, suggesting the existence of a complex of species. Therefore, after the morphological and molecular revision of the material from the different areas, we have confirmed the existence of two species (see below in the Remarks of *T.pulex* sp. nov.).

##### Distribution.

The species has been collected in New Caledonia, French Polynesia and Taiwan. The depth range of the specimens examined is 21– 486 m.

#### ﻿ Trapezionida macilenta sp. nov.

Taxon classificationAnimaliaDecapodaMunididae

8071365D-E6A8-5534-8525-7483655CA8D6

https://zoobank.org/6366F93A-8096-43F6-8FFD-31F15DC11275

Figs 6 , 9A

Trapezionida aff. acola : Machordom et al. 2022: table 2, suppl. figs S1-S6.

##### Material.

***Holotype*: Papua-New Guinea**. Papua Niugini Stn PT05, 3 December 2012, 05°12.4'S, 145°49.3'E, 80 m: male, 5.7 mm (MNHN-IU-2013-1094).

##### Description.

***Carapace***: 1.2× as long as broad, feebly convex, with a few secondary striae and scales between main transverse ridges. Ridges with very short plumose setae and a few scattered long iridescent setae. Intestinal region without scales. Gastric region with five pairs of epigastric spines, longest pair behind supraocular spines, one pair between longest pair; one median protogastric spine. One parahepatic, one anterior branchial, and one postcervical spine on each side. Frontal margins oblique. Lateral margins slightly convex and convergent posteriorly. First lateral spine at anterolateral angle, well-developed, not reaching level of sinus between rostrum and supraocular spines; two or three small spines in front of anterior branch of cervical groove. Branchial margins slightly convex, with five spines. Rostrum spiniform, ~ 0.8× length of remaining carapace, slightly upwards directed, not dorsally carinated. Supraocular spines reaching midlength of rostrum and not reaching end of cornea, subparallel, slightly upwards directed. Pterygostomian region unarmed, ending in round tip.

**Figure 6.**
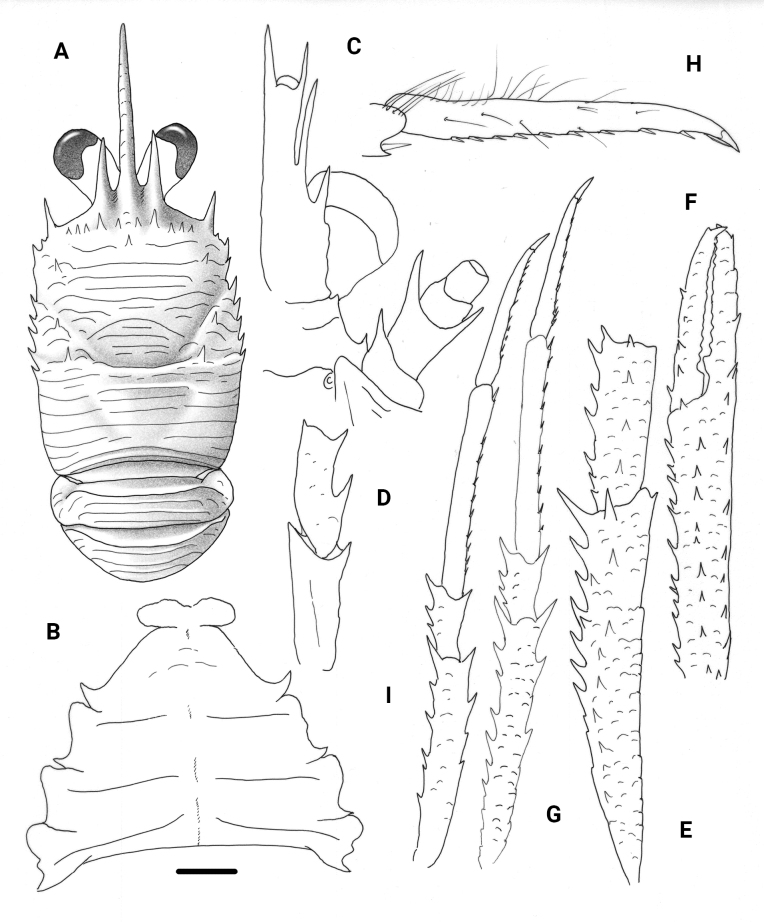
*Trapezionidamacilenta* sp. nov., male holotype, 5.7 mm (MNHN-IU-2013-1094), Papua-New Guinea **A** carapace and pleon, dorsal view **B** sternal plastron **C** cephalic region, showing antennular and antennal peduncles, ventral view **D** right Mxp3 ischium and merus, lateral view **E** right P1, merus and carpus, dorsal view **F** right P1, palm and fingers, dorsal view **G** right P2, lateral view **H** dactylus of right P2, lateral view **I** right P3, lateral view. Scale bars: 1.0 mm (**A, E, F, G, I**); 0.5 mm (**B, C, D, H**).

***Thoracic sternum***: 0.7× as long as broad. Surface of thoracic sternites IV–VI smooth, only a few short scales on sternite IV. Sternite III 3.7× as wide as long. Sternite IV trapezoidal, anterior margin contiguous to sternite III along ¾ of its length; 2.5× as wide as long, and 2.0× as wide as sternite III.

***Pleon***: Ridges of pleomeres unarmed; somites II and III each with three uninterrupted transverse ridges on tergite behind anterior ridge: pleomeres IV and V with anterior ridge only; posteromedian margin of pleomere VI straight.

***Eye***: Ocular peduncle longer than broad. Cornea moderately dilated, maximum corneal diameter 0.3 distance between bases of anterolateral spines.

***Antennule***: Article 1 (distal spines excluded) very long, ~ 0.5× carapace length, 3.0× as long as wide (excluding spines), clearly overreaching end of cornea, with two distal spines, mesial spine longer than lateral; two spines on lateral margin, proximal one short, located at midlength of segment, distal one long, not reaching end of distal spines.

***Antenna***: Article 1 with distomesial spine not exceeding article 2. Article 2 with distomesial spine, exceeding article 3, distolateral spine shorter than distomesial, slightly exceeding article 3. Articles 3 and 4 unarmed.

***Mxp3***: Ischium with well-developed spine on flexor distal margin. Merus slightly shorter than ischium; flexor margin with two spines, median slightly stronger than distal spine; extensor margin with minute distal spine. Carpus unarmed.

***P1***: 4× carapace length, with scattered long plumose setae, and some long iridescent setae; some short setae on spines and scales. Merus 1.5 length of carapace, 2.2× as long as carpus, with some dorsal and mesial spines; distal spines strong, distomesial spine barely reaching proximal fourth of carpus. Carpus 0.6× length of palm, 3.2× as long as broad, with spines along mesial and dorsal sides. Palm 4.4× as long as broad, with row of dorsal spines; one row of spines along mesial and lateral margins, continuing along movable and lateral fingers, respectively. Fingers 0.7× length of palm.

***P2–P3*** (P4 lost): Long and slender, with some short setae and some scattered iridescent setae along extensor margins of all articles. P2 2.8 carapace length. Meri shorter posteriorly (P3 merus 0.9× length of P2 merus); P2 merus as long as carapace, 7.2× as long as broad, 1.2× as long as P2 propodus; P3 merus 6.2× as long as broad, 1.1× as long as P3 propodus. Extensor margins of meri with row of six–eight proximally diminishing spines on P2–P3; flexor margins with three or four spines followed proximally by several eminences; lateral sides unarmed. Carpi with three or four spines on extensor margin; lateral surface with several granules sub-paralleling extensor margin; flexor margin with well-developed distal spine. Propodi 9.0–9.5× as long as broad; extensor margin unarmed; flexor margin with nine or ten slender movable spines, one fixed distal spine. Dactyli slender, length 0.8× that of propodi; flexor margin with ten movable spinules along entire border, with ultimate spinule at base of unguis; P2 dactylus 8.0× as long as wide.

##### Colour in life.

Ground colour of the carapace, pleon and appendages orange with large reddish patches. P1–P4 with reddish and whitish transverse bands. Distal P1 palm reddish (Fig. 9A).

##### Genetic data.

COI, 16S.

##### Etymology.

From the Latin, *macilentus*, thin, in reference to the long and slender antennular peduncle.

##### Remarks.

*Trapezionidamacilenta* belongs to the group of species having one median protogastric spine, anterior ridge of the pleomere II tergite unarmed, thoracic sternites smooth and the antennular article 1 very slender, with the distomesial spine longer than the distolateral spine.

The new species is closely related to *T.diluta* sp. nov. from New Caledonia (see above). However, both species are easily distinguished by several characters:

- The branchial lateral margin of the carapace has four spines in *T.diluta* and five in *T.macilenta*. The pleomeres II and III tergites each with two or three uninterrupted transverse ridges behind the anterior ridge *T.macilenta*, whereas there is only one transverse ridge in *T.diluta*.
- The extensor margin of the Mxp3 merus has one minute distal spine in *T.macilenta*, absent in *T.diluta*.
- Genetically both species are different. *T.macilenta* showed high divergence values compared with *T.diluta* (6.42% COI, 4.6% 16S).

##### Distribution.

Papua-New Guinea, 80 m depth.

#### ﻿ Trapezionida microtes sp. nov.

Taxon classificationAnimaliaDecapodaMunididae

27D3E523-FAE8-5B72-91BD-A4F17FA680C8

https://zoobank.org/AB42F369-2D53-4AB1-89FD-EEA3C6083348

Fig. 7

Trapezionida pumila : Machordom et al. 2022: table 2, suppl. figs S1–S6 (non T.pumilla Macpherson, 2004).

##### Material.

***Holotype***: Vanuatu. Santo Stn AT9, 17 September 2006, 15°41.5'S, 167°01.3'E, 481 m: ovigerous female, 3.4 mm (MNHN-IU-2017-1337). ***Paratypes***: Philippines. Musorstom 2 Stn DG32, 24 November 1980, 13°40'N, 120°54'E, 192–220 m: 1 male, 2.0 mm, 2 ovigerous females, 2.3–2.4 mm, 1 female, 2.1 mm (MNHN-IU-2014-13975). New Caledonia. Halipro 2 Stn BT94, 24 November 1996, 23°33'S, 167°42'E, 448–880 m: 1 male, 3.0 mm (MNHN-IU-2014-13978). — Lithist Stn DW1, 10 August 1999, 23°37.4'S, 167°42.07'E, 440 m: 1 ovigerous female, 3.3 mm (MNHN-IU-2014-13979).

##### Description.

***Carapace***: Slightly longer than broad, with a few scales between main transverse ridges in epigastric and anterior branchial areas. Ridges with very short setae. Gastric region with five pairs of epigastric spines, longest pair behind supraocular spines, one pair between longest pair. One parahepatic spine on each side. Frontal margins transverse. Lateral margins subparallel and convergent posteriorly. First lateral spine at anterolateral angle, well-developed, not reaching level of sinus between rostrum and supraocular spines. Branchial margin with four small spines. Rostrum slightly triangular, ~ 0.5× length of remaining carapace, slightly upwards directed, dorsally carinated. Supraocular spines short, not reaching midlength of rostrum and clearly not reaching end of cornea, subparallel. Pterygostomian region unarmed, ending in a round tip.

**Figure 7.**
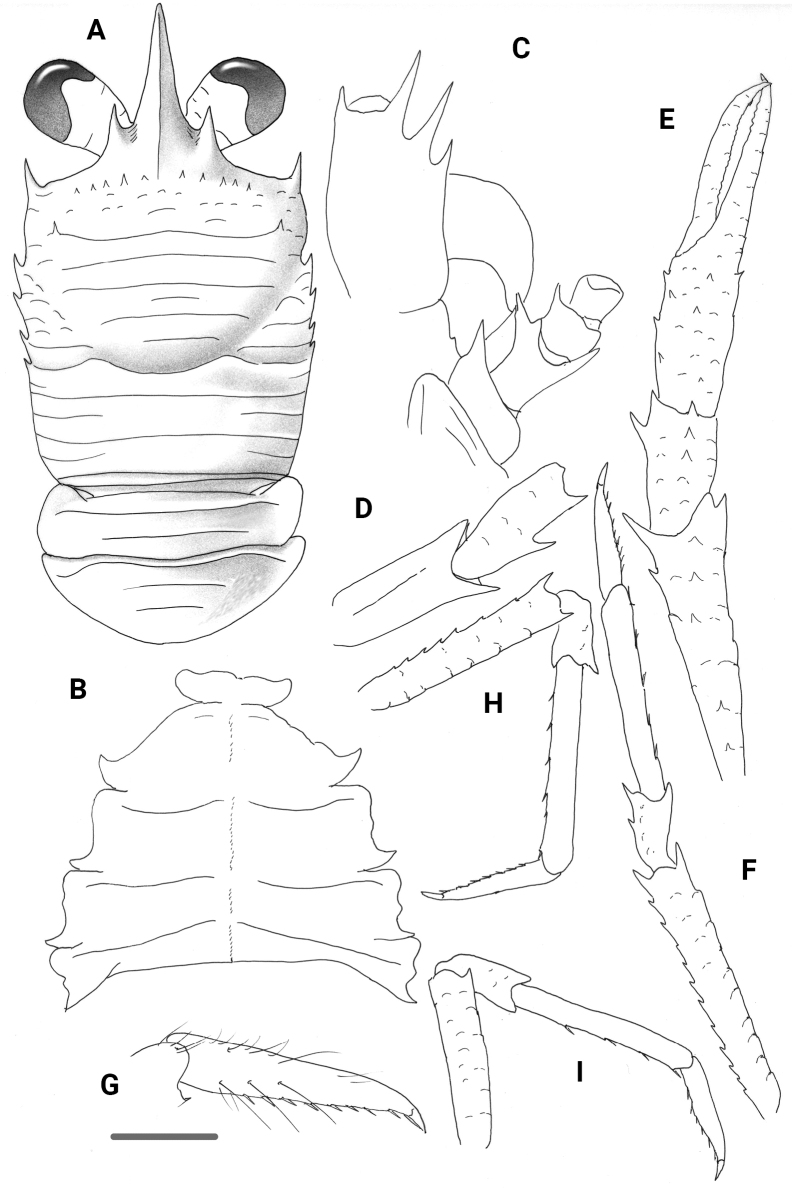
*Trapezionidamicrotes* sp. nov., ovigerous female holotype, 3.4 mm (MNHN-IU-2017-1337), Vanuatu **A** carapace and pleon, dorsal view **B** sternal plastron **C** cephalic region, showing antennular and antennal peduncles, ventral view **D** right Mxp3 ischium and merus, lateral view **E** right P1, dorsal view **F** right P2, lateral view **G** dactylus of right P2, lateral view **H** right P3, lateral view **I** right P4, lateral view. Scale bars: 1.0 mm (**A, E, F, H, I**); 0.5 mm (**B, C, D, G**).

***Thoracic sternum***: 0.8× as long as wide. Surface of thoracic sternites IV–VI smooth, only a few short scales on sternite IV. Sternite III 3.5× as wide as long. Sternite IV trapezoidal, anterior margin contiguous to sternite III along ¾ of its length; 2.5× as wide as long, and 2.3× as wide as sternite III.

***Pleon***: Ridges of pleomeres unarmed; tergites II and III each with one or two uninterrupted transverse ridges on tergite behind anterior ridge: tergites IV and V with anterior ridge only; posteromedian margin of tergite VI straight.

***Eye***: Ocular peduncle longer than broad, cornea dilated, maximum corneal diameter 0.3 distance between bases of anterolateral spines.

***Antennule***: Article 1 (distal spines excluded) ~ 0.3× carapace length, 2.0× as long as wide (excluding spines), not overreaching end of cornea, with two distal spines, mesial spine shorter than lateral; two spines on lateral margin, proximal one short, located at midlength of segment, distal one long, not reaching end of distolateral spines.

***Antenna***: Article 1 with short distomesial spine not exceeding end of article 2. Article 2 with distomesial and distolateral subequal spins, not reaching end of article 3. Article 3 with distomesial spine.

***Mxp3***: Ischium with well-developed spine on flexor distal margin. Merus slightly shorter than ischium; flexor margin with two spines, median slightly stronger than distal spine; extensor margin with minute distal spine. Carpus unarmed.

***P1***: 2.5× carapace length, with scattered long setae and some short setae on spines and scales. Merus 0.9× carapace length, 2.0× as long as carpus, with some dorsal and mesial spines; distal spines strong, distomesial spine barely reaching proximal fourth of carpus. Carpus as long as palm, 2.0× as long as broad, with spines along mesial and dorsal sides. Palm 2.0× as long as broad, with row of minute dorsal spines; one row of spines along mesial and lateral margins. Fingers unarmed, 1.3× length of palm.

***P2–P4***: Long and slender, with some short setae and some scattered iridescent setae along extensor margins of all articles. P2 2.2× carapace length. Meri shorter posteriorly (P3 merus 0.9× length of P2 merus, P4 merus 0.7× length of P3 merus); P2 merus 0.9× carapace length, 6.0× as long as broad, 1.4× as long as P2 propodus; P3 merus 5.5× as long as broad, 1.2× as long as P3 propodus. Extensor margins of meri with row of 10–12 proximally diminishing spines on P2–P3, only distal spine on P4; flexor margins with three or four spines followed proximally by several eminences on P2–P3, only distal spine on P4; lateral sides unarmed. Carpi with one or two spines on extensor margin; lateral surface with several granules sub-paralleling extensor margin; flexor margin with well-developed distal spine. Propodi 6.0 (P2), 5.5 (P3), 4.2 (P4) × as long as broad; extensor margin unarmed; flexor margin with four or five five slender movable spines, one fixed minute distal spine. Dactyli slender, length 0.6–0.7 that of propodi; flexor margin with eight or nine movable spinules along entire border, with ultimate spinule at base of unguis; P2 dactylus 5.0× as long as wide. P4 merocarpal articulation reaching anterior end of cervical groove; P4 merus > ½ length of P2 merus.

##### Genetic data.

COI, 16S.

##### Etymology.

From the Greek, *mikros*, small, in reference to the small size of the species.

##### Remarks.

*Trapezionidamicrotes* belongs to the group of species having four spines on the branchial lateral margins of the carapace, rostrum narrowly triangular (not spiniform), short supraocular spines, thoracic sternites smooth, moderately large eyes, pleomere II tergite unarmed, and the distomesial spine of the antennular article 1 smaller than the distolateral spine.

The new species is closely related to *T.alonsoi* (Macpherson, 1994) from New Caledonia area and *T.pumilla* (Macpherson, 2004) from Tonga. However, *T.microtes* is easily distinguished from these species by several characters:

- The branchial lateral margin has four spines in the new species, whereas it is armed with five spines (rarely three or four) in *T.alonsoi* and *T.pumilla*.
- The extensor margin of the Mxp3 merus has a distal spine in *T.alonsoi* and *T.pumilla*, whereas it is unarmed in the new species.
- The P2–P4 dactyli are clearly more slender in *T.microtes* than in *T.alonsoi*: 5.0× vs. 2.5× as long as wide.
- Genetically *T.microtes* showed high divergence values with *T.alonsoi* (6.43% COI, 3.77% 16S). No genetic data is available for *T.pumilla*.

The new species is also close to *T.trigonocornus* from Japan (Komai 2012). However, the dorsal surface of the carapace has branchial dorsal and postcervical spines in *T.trigonocornus*, whereas these spines are absent in the new species. No genetic data is available for *T.trigonocornus*.

##### Distribution.

Vanuatu, New Caledonia, at 192–880 m.

#### ﻿ Trapezionida pulex sp. nov.

Taxon classificationAnimaliaDecapodaMunididae

E9DC28F4-DBFF-5F60-9CC6-D12159ED4444

https://zoobank.org/FF992C41-5EC1-4109-9206-EB62CB87243B

Figs 8 , 9B

Munida leptitis : Macpherson 1996: 394, fig. 14. — Macpherson 1997: 607. — Macpherson 2000: 419. — Macpherson 2004: 263. — Baba 2005: 267 (in part). — Macpherson 2006: 318 (in part). — Baba et al. 2008: 104 (in part). — Macpherson 2013: 301 (in part). — Macpherson et al. 2020: 59. (non M.leptitis Macpherson, 1994).
Trapezionida aff. *leptitis1*: Machordom et al. 2022: table 2, suppl. figs S1–S6.

##### Material.

***Holotype***: New Caledonia, Exbodi Stn DW3902, 19°53'S, 165°49'E, 410 m, 22 September 2011: male, 5.4 mm (MNHN-IU-2016-5809). ***Paratypes***: Indonesia, Kei Islands, Karubar Stn DW02, 22 October 1991, 05°47'S, 132°13'E, 209–240 m: 3 males, 4.8–5.3 mm, 2 females, 3.6–4.3 mm, 1 juv. 2.4 mm (MNHN-IU-2014-14752, MNHN-IU-2016-516). Papua-New Guinea, Biopapua Stn CP3759, 14 October 2010, 03°59.690'S, 153°37.070'E, 287–352 m: 1 ovigerous female, 5.3 mm (MNHN-IU-2011-3829). — Papua Niugini Stn CP4016, 12 December 2012, 05°40'S, 148°14'E, 280–285 m: 1 female, 3.2 mm (MNHN-IU-2016-5816). — Madeep Stn DW4310, 3 May 2014, 09°50'S, 151°31'E, 390–500 m: 1 male, 4.2 mm (MNHN-IU-2016-5804). — Stn DW4311, 3 May 2014, 09°50'S, 151°32'E, 270–486 m: 1 male, 5.7 mm, 1 ovigerous female, 5.2 mm (MNHN-IU-2015-828). — Kavieng 2014 Stn DW4485, 5 September 2014, 02°26'S, 149°54'E, 240–242 m: 1 male, 3.4 mm (MNHN-IU-2014-9905). Solomon Islands, Salomon 1 Stn CP1831, 05 October 2001, 10°12.1'S, 161°19.2'E, 135–325 m: 1 ovigerous female, 6.0 mm; (MNHN-IU-2016-2997), 3 males, 3.4–5.5 mm, 1 ovigerous female, 3.7 mm, 1 female, 5.0 mm (MNHN-IU-2014-14750). Vanuatu, Musorstom 8 Stn CP983, 23 September 1994, 19°22'S, 169°28'E, 475–480 m: 1 male, 3.5 mm (MNHN-IU-2016-515). New Caledonia, Chesterfield Islands, Ebisco Stn CP2620, 20 October 2005, 20°05.864'S, 160°22.318'E, 270–532 m: 1 female, 5.8 mm (MNHN-IU-2014-14714). — Kanadeep 1 Stn DW5007, 22°12'S, 159°02'E, 19 September 2017, 290–750 m: 1 male, 5.6 mm (MNHN-IU-2013-19924). New Caledonia, Terrasse Stn DW3083, 24 October 2008, 22°27'S, 167°25'E, 470–570 m: 1 male, 5.2 mm (MNHN-IU-2011-4956). — Exbodi Stn CP3927, 26 September 2011, 18°36'S, 164°20'E, 381 m: 1 ovigerous female, 4.7 mm (MNHN-IU-2016-5815). — Stn DW3928, 26 September 2011, 18°38'S, 164°20'E, 362–402 m: 2 males, 4.5–5.2 mm (MNHN-IU-2016-5811). — Stn DW3930, 26 September 2011, 18°37'S, 164°26'E, 448–464 m: 2 males, 5.3–6.0 mm (MNHN-IU-2011-6466). — Stn DW3937, 27 September 2011, 18°37'S, 164°26'E, 446–604 m: 1 male, 7.8 mm (MNHN-IU-2013-1793), 2 males, 4.2–6.0 mm, 2 ovigerous females, 5.2–6.0 mm, 1 female, 3.8 mm(MNHN-IU-2011-7263). — Stn CP3841, 10 September 2011, 22°23'S, 167°24'E, 477–503 m: 2 ovigerous females, 4.8-5.3 mm (MNHN-IU-2016-5819). — Stn DW3847, 13 September 2011, 22°04'S, 168°41'E, 414–435 m: 1 ovigerous female, 4.4 mm (MNHN-IU-2013-1882). — Stn CP3848, 13 September 2011, 22°03'S, 168°42'E, 430–440 m: 3 males, 4.2–5.1 mm, 4 ovigerous females, 4.4–4.7 mm (MNHN-IU-2014-18231). — Stn CP3849, 13 September 2011, 22°03'S, 168°41'E, 360–560 m: 1 male, 6.0 mm (MNHN-IU-2016-5812). — Kanacono Stn DW4764, 27 August 2016, 23°20'S, 168°15'E, 350–356 m: 1 male, 7.0 mm (MNHN-IU-2017-8561). Wallis and Futuna Islands, Musorstom 7 Stn DW605, 13°21.3'S, 176°08.4'W, 335–340 m, 26 June 1992: 1 ovigerous female, 4.5 mm (MNHN-IU-2016-517), 7 males, 3.1–6.1 mm, 3 ovigerous females, 4.4–5.3 mm, 2 females, 3.6–4.0 mm (MNHN-IU-2014-14735). Tonga, Bordau 2 CP1545, 5 June 2000, 21°17'S, 17517°W, 444–447 m: 3 males, 4.1–6.2 mm, 1 ovigerous female, 6.0 mm (MNHN-IU-2014-14739). — Stn CP1643, 21°04.54'S, 175°22.50'W, 487 m, 22 June 2000: 2 males, 5.7–6.8 mm (MNHN-IU-2014-14748), 1 female, 4.6 mm (MNHN-IU-2016-518).

**Figure 8.**
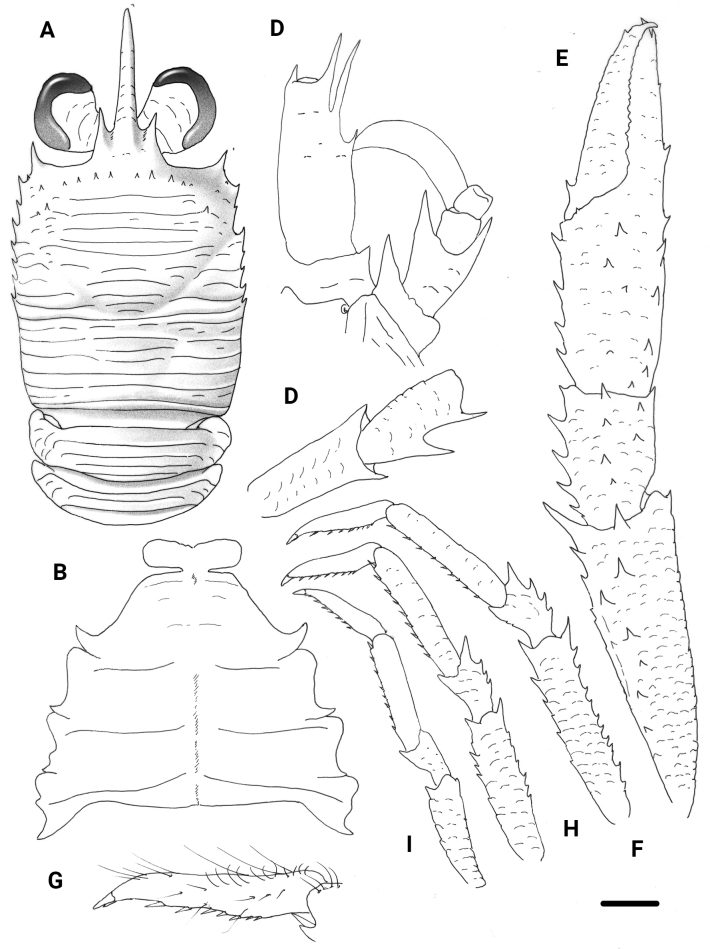
*Trapezionidapulex* sp. nov., male holotype, 5.4 mm (MNHN-IU-2016-5809), New Caledonia **A** carapace and pleon, dorsal view **B** sternal plastron **C** cephalic region, showing antennular and antennal peduncles, ventral view **D** right Mxp3 ischium and merus, lateral view **E** right P1, dorsal view **F** left P2, lateral view **G** dactylus of left P2, lateral view **H** left P3, lateral view **I** left P4, lateral view. Scale bars: 1.0 mm (**A, E, F, H, I**); 0.5 mm (**B, C, D, G**).

##### Description.

***Carapace***: Slightly longer than broad, feebly convex, with some secondary striae and scales between main transverse ridges. Dorsal ridges with dense short setae, and numerous scattered, long, non-iridescent setae. Gastric region with four or five pairs of epigastric spines, longest pair behind supraocular spines, with intermediate pair of minute spines between large epigastric pair. One or two small hepatic and one parahepatic spine on each side, branchial dorsal and postcervical spines absent. Frontal margins transverse. Lateral margins slightly convex. First lateral spine on anterolateral angle, short, clearly not reaching level of sinus between rostrum and supraocular spines; two small spines in front of anterior branch of cervical groove; end of anterior branch of cervical groove with tuft of iridescent setae. Branchial margins straight, with five small spines, decreasing in size posteriorly. Rostrum spiniform, ~ 0.6–0.7× length of remaining carapace, laterally carinate, slightly upwards directed. Supraocular spines short, not reaching midlength of rostrum and clearly not reaching end of cornea, subparallel, directed slightly upwards. Grooves between rostrum and supraocular spines moderately shallow. Pterygostomian region unarmed, ending in round tip.

***Thoracic sternum***: Approximately 0.7× as long as wide. Maximum width of sternum at sternite VII. Surface of thoracic IV–VI sternites smooth, with a few short striae in sternite IV. Sternite IV trapezoidal; anterior margin wide and subparallel to sternite III along its entire length. Sternite III ~ 2.5–3.0× as wide as long, sternite IV 2.5× as wide as long, 2.5× as wide as sternite III.

***Pleon***: Tergites II and III each unarmed along anterior ridge, with three uninterrupted transverse ridges on tergite behind anterior ridge, tergites IV and V each with two uninterrupted transverse ridges; ridges with some short setae and a few iridescent setae.

***Eye***: Cornea dilated, much wider than peduncle. Maximum corneal diameter 0.4 distance between bases of anterolateral spines.

***Antennule***: Article 1 more than 2.0× as long as wide, with two well-developed distal spines, distomesial spine clearly shorter than distolateral; two lateral spines, distal much longer than proximal and nearly exceeding distolateral spine.

***Antenna***: Article 1 with short distomesial spine nearly reaching end of article 2. Article 2 with distomesial and distolateral spines exceeding end of article 3. Article 3 unarmed.

***Mxp3***: Ischium with strong distal spine on flexor margin. Merus shorter than ischium; flexor margin with two spines, proximal stronger than distal; extensor margin unarmed or with minute spine. Carpus unarmed.

***P1***: 2.5–2.7 (females), 3.0–3.2 (males) × carapace length, squamate, covered with numerous long plumose and iridescent setae along mesial margin of articles. Merus 1.0–1.2 length of carapace, 2.1–2.2× as long as carpus, with some dorsal and mesial spines; distal spines strong, distomesial spine nearly reaching proximal third of carpus. Carpus 0.9–1.0 length of palm, 1.4–1.6× as long as broad, with strong spines along mesial margin, some small spines on dorsal side. Palm 1.3–1.7× as long as broad, with some small dorsal spines; well-developed spines along lateral and mesial margins. Fingers 1.2–1.4× as long as palm; fixed finger with spines along lateral margin; movable finger with basal and distal spines.

***P2–P4***: Moderately long and slender, with numerous iridescent setae along extensor margin of articles. P2 1.9–2.0× carapace length. Meri shorter posteriorly (P3 merus 0.8–0.9 length of P2 merus, P4 merus 0.7–0.8 length of P3 merus); P2 merus 0.7–0.8 length of carapace, 4.8–4.9× as long as broad, 1.3–1.4× as long as P2 propodus; P3 merus 4.8–4.2× as long as broad, 1.2–1.3× as long as P3 propodus; P4 merus 3.5–3.7× as long as broad, 1.1–1.2× length of P4 propodus. Extensor margins of P2–P4 meri with row of spines, decreasing in size proximally; flexor margins with some well-developed spines followed proximally by several eminences; lateral sides unarmed. Carpi with three or four spines on extensor margin of P2–P3, unarmed on P4; lateral surface with several granules sub-paralleling extensor margin on P2–P4; flexor margin with distal spine. Propodi 4.0–4.7× as long as broad; extensor margin unarmed; flexor margin with nine or ten slender movable spines on P2–P4, distal end with one fixed spine. Dactyli slender, slightly shorter than propodi; flexor margin with 7–9 movable spinules, with ultimate spinule at base of unguis, penultimate spine much closer to antepenultimate than to ultimate spine; P2 dactylus 3.7–4.5× as long as wide. P4 merocarpal articulation not reaching anterior end of cervical groove; P4 merus ~ ½ length of P2 merus.

##### Colour in life.

**Figure 9.**
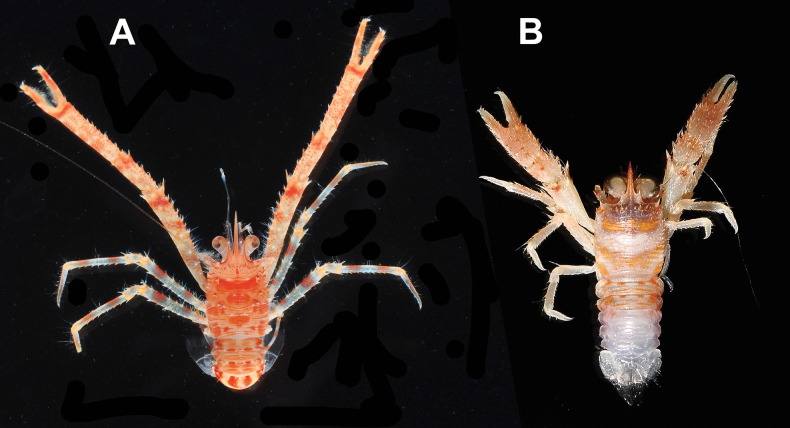
Colour in life **A***Trapezionidamacilenta* sp. nov., male holotype, 5.7 mm (MNHN-IU-2013-1094), Papua-New Guinea **B***Trapezionidapulex* sp. nov., ovigerous female paratype, 4.4 mm (MNHN-IU-2013-1882), New Caledonia.

Ground colour of carapace and pleomere tergites pale orange. Rostrum orange, supraocular spines whitish. Transverse whitish stripes on median and posterior parts of carapace. Median and lateral parts of pleomere tergites with orange spots. P1–P4 pale orange without transverse stripes; P1 with a few dorso-median red spots; distal portion of fingers whitish. P2–P4 dactyli whitish (Fig. 9B).

##### Genetic data.

COI, 16S.

##### Etymology.

From the Latin, *pulex*, flea, in reference to the small size of the species.

##### Remarks.

The new species is morphologically very close to *T.leptitis* (Macpherson, 1994) (see above), whichwas described from specimens collected in New Caledonia, and it was found later off Wallis and Futuna area, Taiwan, Indonesia, Papua-New Guinea, Vanuatu, Fiji, Tonga, and French Polynesia (see Macpherson et al. 2020 and references cited therein). Nevertheless, some slight morphological differences among specimens suggest the existence of a complex of cryptic species with overlapping distribution ranges. The molecular analysis of specimens from the different localities has demonstrated the existence of two species, *T.leptitis* and *T.pulex*. Both species are genetically distinct, although only separated morphologically by slight differences.

Both species can be differentiated by the following aspects:

- The thoracic sternite III is more slender in *T.leptitis* than in *T.pulex* (3.5–4.5× as wide as long in *T.leptitis* and 2.5–3.0× in *T.pulex*).
- The P2–P4 are more slender in *T.pulex* than in *T.leptitis*. The propodi are 5.3–5.7× as long as broad and the dactyli 5.0× as long as broad in *T.pulex*. These values are 4.0–4.6 and 3.7–4.5×, respectively, in *T.leptitis* .
- The colour patterns of both species are different. For instance, the dorsal surface of the P1 has numerous red spots in *T.leptitis*, whereas these spots are very few in *T.pulex*.
- Genetically the new species is different from *T.leptitis*: 7.44% 16S and 13.54% COI.

##### Distribution.

The species is found in Indonesia, Vanuatu, Loyalty Islands, New Caledonia, Wallis and Futuna Islands, Fiji, Tonga, and French Polynesia. The depth range of these specimens is 135–750 m.

### ﻿Genus *Typhlonida* Macpherson & Baba, 2022 in Machordom et al. 2022

#### ﻿ Typhlonida eluminata sp. nov.

Taxon classificationAnimaliaDecapodaMunididae

6DF9648E-EE89-535C-A8F3-0B055A5C081E

https://zoobank.org/DC2ED9D2-2F9E-4C5D-B3CA-C24513DA6109

Fig. 10

Typhlonida sp.: Machordom et al. 2022: fig. 3H, table 2.

##### Material.

***Holotype***: New Caledonia, Exbodi Stn DW3941, 19°04'S, 164°03'E, 980-1090 m, 28 September 2011: female, 6.6 mm (MNHN-IU-2011-6787).

##### Description.

***Carapace***: Slightly longer than broad, feebly convex, with a few secondary striae between main transverse ridges and some scales on gastric and anterior branchial areas. Dorsal ridges with dense short plumose setae, and a few scattered long setae. Gastric region with two pairs of epigastric spines, longest pair behind supraocular spines. Parahepatic, branchial dorsal and postcervical spines absent. Frontal margins slightly oblique. Lateral margins slightly convex. First lateral spine at anterolateral angle, well-developed, reaching level of sinus between rostrum and supraocular spines; one small spine in front of anterior branch of cervical groove. Branchial margins slightly convex, with five spines, decreasing in size posteriorly. Rostrum spiniform, ~ 0.5× length of remaining carapace, horizontal. Supraocular spines reaching midlength of rostrum and exceeding end of cornea, subparallel, directed slightly upwards. Pterygostomian region ending in round tip.

***Thoracic sternum***: Approximately 0.7× as long as wide. Surface of thoracic IV–VI sternites smooth, with a few short striae in sternite IV. Sternite III ~ 4× as wide as long. Sternite IV triangular, anterior margin clearly narrower than preceding sternite, anterolateral margins slightly convex; 2.0× as wide as long, 2.3× as wide as sternite III.

***Pleon***: Pleomere II tergite with three–four pairs of spines along anterior ridge, with one uninterrupted transverse ridge on tergite behind anterior ridge; tergites III and IV each with additional ridge behind anterior ridge; ridges with some short setae and a few iridescent setae.

***Eye***: Cornea not dilated, as wide as peduncle. Maximum corneal diameter < 0.3× distance between bases of anterolateral spines.

***Antennule***: Article 1 (basal) 0.7× as wide as long, with two well-developed distal spines, distomesial spine shorter than distolateral; two lateral spines, distal much longer than proximal and not reaching distolateral spine.

***Antenna***: Article 1 with short distomesial spine reaching end of article 2. Article 2 with subequal distomesial and distolateral spines not reaching end of article 3; article 3 with well-developed distomesial spine.

***Mxp3***: Ischium with strong distal spine on flexor margin. Merus shorter than ischium; flexor margin with two well-developed spines, proximal stronger than distal; extensor margin unarmed. Carpus unarmed.

***P1***: 2.2× carapace length, with dense long setae along mesial and dorsal margins of articles, more numerous in paratypes than in holotype. Merus 0.9× length of carapace, 2.1× as long as carpus, with some dorsal and mesial spines; distal spines strong, distomesial spine nearly reaching proximal 1/3 of carpus. Carpus 0.9× length of palm, 2.0× as long as broad, with strong spines along mesial margin, some small spines on dorsal side. Palm 2.0× as long as broad, with row of small dorsal spines; well-developed spines along lateral and mesial margins. Fingers 1.2× as long as palm; movable finger unarmed, fixed finger with distal spine.

***P2–P4***: Moderately long and slender, with some plumose setae and scattered longer setae along extensor margin of articles. P2 2.0× carapace length. Meri successively shorter posteriorly (P3 merus 0.9× length of P2 merus, P4 merus 0.7× length of P3 merus); P2 merus 0.8× length of carapace, 5.2× as long as broad, 1.5× as long as P2 propodus; P3 merus 5.0× as long as broad, 1.3× as long as P3 propodus; P4 merus 4.0× as long as broad, 1.2× length of P4 propodus. Extensor margins of P2–P4 meri with row of spines, decreasing in size proximally; flexor margins with well-developed distal spine followed proximally by several spines or eminences; lateral sides unarmed. Carpi with distal spine on extensor margin of P2–P4; lateral surface with several granules sub-paralleling extensor margin on P2–P4; flexor margin with distal spine. Propodi 7.5 (P2) to 6.0 (P4) × as long as broad; extensor margin unarmed; flexor margin with five or six slender movable spines on P2–P4, distal end with one fixed spine. Dactyli slender, 0.6–0.7× as long as propodi; flexor margin with seven–eight movable spinules, with ultimate spinule at base of unguis, penultimate spine equidistant between antepenultimate and ultimate spines; P2 dactylus 4.5× as long as wide.

**Figure 10.**
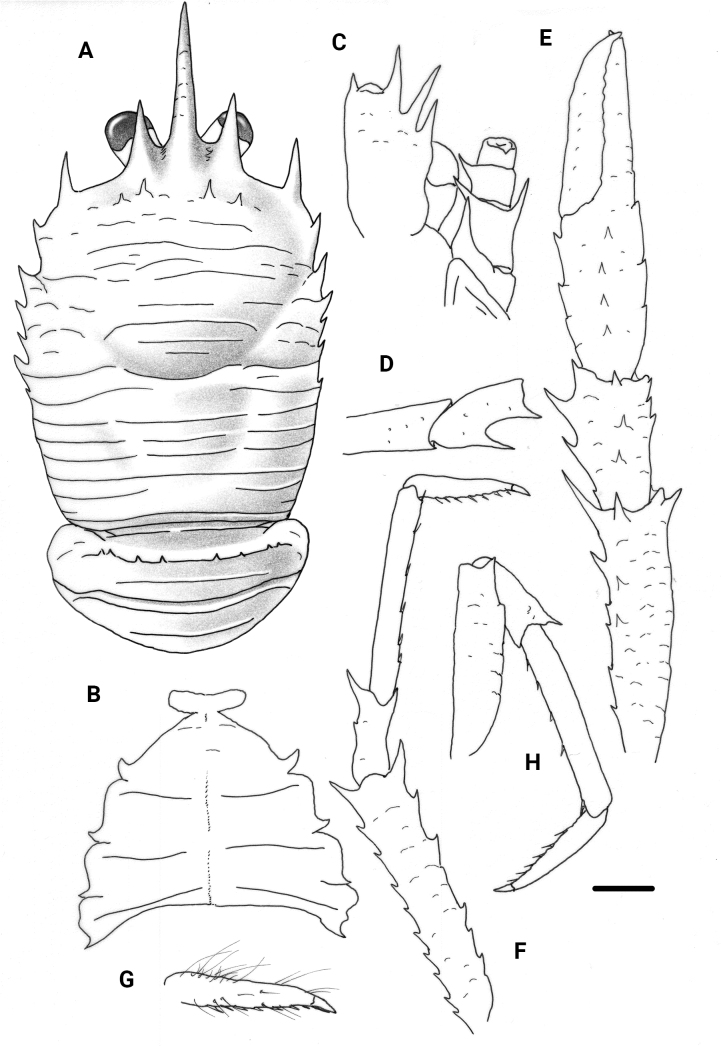
*Typhlonidaeluminata* sp. nov., female holotype, 6.6 mm (MNHN-IU-2011-6787), New Caledonia **A** carapace and pleon, dorsal view **B** sternal plastron **C** cephalic region, showing antennular and antennal peduncles, ventral view **D** right Mxp3 ischium and merus, lateral view **E** right P1, dorsal view **F** right P2, lateral view **G** dactylus of right P2, lateral view **H** right P4, lateral view. Scale bars: 1.0 mm (**A, E, F, H**); 0.6 mm (**B, C, D, G**).

##### Genetic data.

COI, 16S, 18S.

##### Etymology.

From the Latin, *eluminatus*, blinded, in reference to the small eyes.

##### Remarks.

*Typhlonidaeluminata* belongs to the group of species having five minute spines on the branchial lateral margins of the carapace, short supraocular spines, eyes small, cornea as wide as peduncle, maximum corneal diameter < 0.3× distance between bases of anterolateral spines, the anterior ridge of the pleomere II tergite with spines and the distomesial spine of the antennal article 1 well-developed, exceeding midlength of article 2. The new species is closely related to *T.typhle* (Macpherson, 1994), from New Caledonia and *T.galalala* (McCallum et al., 2021) from NW Australia.

The new species can be distinguished from *T.typhle* by the presence of a distomesial spine on the antennal article 3 in the new species, which is absent in *T.typhle*. Furthermore, the anterolateral spine of the carapace clearly not reaching the level of the sinus between the rostrum and the supraocular spines in *T.typhle*, whereas this spine is reaching this sinus in the new species. Finally, the flexor margin of the Mxp3 merus has two well-developed spines in the new species, whereas there is only one median spine in *T.typhle*.

The differences between *T.typhle* and *T.galalala* are the following:

- The anterolateral spine of the carapace is as long as the supraocular spine in the new species, whereas it is smaller in *T.galalala*.
- The P2 merus is longer than the carapace in *T.galalala*, whereas it is shorter in the new species.
- The dorsal surface of the P1 palm is unarmed in *T.galalala*, whereas there are some small spines in the new species.
- Genetically the new species is different from *T. typhle and T. galalala*: *T.eluminata* diverges respect to *T.typhle* 3.97% for 16S and 11.42% for COI, and the values are ~ 11.61% for COI and 3.86% for 16S compared to *T.galalala*.

The new species is also close to *T.lanciaria* (Cabezas et al., 2011), from Taiwan, but this species has the cornea clearly wider than the peduncle. Furthermore, both species are genetically quite different (3.01% for 16S) and 13. 4% (COI).

##### Distribution.

New Caledonia, between 980 and 1090 m.

## Supplementary Material

XML Treatment for Garymunida namora

XML Treatment for Trapezionida brachytes

XML Treatment for Trapezionida brevitas

XML Treatment for Trapezionida diluta

XML Treatment for Trapezionida leptitis

XML Treatment for Trapezionida macilenta

XML Treatment for Trapezionida microtes

XML Treatment for Trapezionida pulex

XML Treatment for Typhlonida eluminata

## ﻿Additional information

### Author ORCIDs

Enrique Macpherson https://orcid.org/0000-0003-4849-4532

Paula C. Rodríguez-Flores https://orcid.org/0000-0003-1555-9598

Annie Machordom https://orcid.org/0000-0003-0341-0809

### Data availability

All of the data that support the findings of this study are available in the main text or Supplementary Information.

## Supplementary materials

Supplementary material 1 Dichotomous keys to species of the three genera - *Garymunida, Trapezionida* and *Typhlonida* Data type docx Explanation note Key to species of the genus *Garymunida* Macpherson & Baba, in Machordom et al. 2022. Key to species of the genus *Trapezionida* Macpherson & Baba, in Machordom et al. 2022. Key to species of the genus *Typhlonida* Macpherson & Baba, in Machordom et al. 2022. https://binary.pensoft.net/file/961274This dataset is made available under the Open Database License (http://opendatacommons.org/licenses/odbl/1.0/). The Open Database License (ODbL) is a license agreement intended to allow users to freely share, modify, and use this Dataset while maintaining this same freedom for others, provided that the original source and author(s) are credited.Enrique Macpherson, Paula C. Rodríguez-Flores, Annie Machordom
