# Supplementary material for: ﻿DNA barcoding and morphology revealed the existence of seven new species of squat lobsters in the family Munididae (Decapoda, Galatheoidea) in the southwestern Pacific
Source: Zookeys. 2024 Jan 3;1188:91–123. doi: 10.3897/zookeys.1188.114984 (PMC10782442; doi:10.3897/zookeys.1188.114984)
Supplement: Supplementary material 1 — Dichotomous keys to species of the three genera - Garymunida, Trapezionida and Typhlonida [file zookeys-1188-091_article-114984__-s001.docx]

**SUPLEMENTARY MATERIAL**

**Key to species of the genus *Garymunida* Macpherson & Baba, in Machordom et al. 2022**

1. Supraocular spines overreaching rostral tip 2

*—* Supraocular spines falling short of rostral tip 3

2. Pleomere 4 tergite lacking spine on posterior transverse ridge

*G. longipes* (A. Milne Edwards, 1880)

— Pleomere 4 tergite armed with spine on posterior transverse ridge

*G. longispinata* (Baba, 1988)

3. Dorsal carapace surface with numerous minute spines

*G. schroederi* (Chace, 1939)

— Dorsal carapace surface with a few well developed spines 4

4. Pleomere 4 tergite lacking spine on posterior transverse ridge 5

— Pleomere 4 tergite armed with spine on posterior transverse ridge 9

5. Two spines on posterior-most transverse ridge of carapace 6

— No spine on posterior-most transverse ridge of carapace 8

6. Mxp 3 merus with distal spine on extensor margin. Distomesial spines of antennal articles 2–3 exceeding far beyond end of peduncle. One postcervical spine on each side *G. laurentae* (Macpherson, 1994)

— Mxp 3 merus unarmed on extensor margin. Distomesial spines of antennal articles 2–3 terminating in end of peduncle. Two postcervical spines on each side 7

7. Frontal margin oblique. Sternal plastron with numerous striae

*G. ocyrhoe* (Macpherson, 1994)

— Frontal margin transverse. Sternal plastron with striae moderate in density

*G. pilosimanus* (Baba, 1969)

8. Mxp 3 merus with distal spine on extensor margin. *G. sabatesae* (Macpherson, 1994)

— Mxp 3 merus unarmed on extensor margin. *G. aequabilis* (Macpherson, 2006)

9. Pair of protogastric spines behind pair of epigastric spines 10

— No protogastric spines 13

10. Cardiac región unarmed. Posterior margin of carapace with 2 median spines

***G. namora*** sp. nov.

— Cardiac región some spines. Posterior margin of carapace with 6 spines 11

11. P2–4 propodi 18–19 times as long as broad *G. procera* (Ahyong & Poore, 2004)

— P2–4 propodi 12–13 times as log as broad 12

12. P2-4 propodi 3 times dactylus length *G. imitata* (Macpherson, 2006)

— P2-4 propodi 2 times dactylus length *G. soelae* (Baba, 1986)

13. Branchial lateral margin with 3 spines 14

— Branchial lateral margin with 4 spines 16

14. Posterior margin of carapace unarmed *G. isabelensis* (Cabezas et al., 2009)

— Posterior margin of carapace with 2 median spines 15

15. Distomesial spine of antennular article 1 larger than distolateral spine

*G. nielbrucei* (Vereshchaka, 2005)

— Distomesial spine of antennular article 1 shorter than distolateral spine

*G. prolixa* (Alcock, 1894)

16. Transverse row of small spines on cardiac region (rarely absent) 17

— Prominent median spine on cardiac region 18

17. Distomesial spine of second antennal segment overreaching third segment. Parahepatic spine on each side of carapace *G. simillina* (Macpherson, 2006)

— Distomesial spine of second antennal segment never reaching end of third segment. Parahepatic spine usually absent  *G. normani* (Henderson, 1885)

18. Posterior margin of carapace unarmed 19

— Posterior margin of carapace with 2 median spines 20

19. Sternites 4–6 smooth, without striae. *G. garciai* (Macpherson, 2004)

— Sternites 4–6 with striae. *G. similis* (Baba, 1988)

20. Article 2 of antennal peduncle with distinct spine on distomesial margin

*G. squamosa* (Henderson, 1885)

— Article 2 of antennal peduncle unarmed on distomesial margin

*G. analoga* (Macpherson, 1993)

**Key to species of the genus *Trapezionida* Macpherson & Baba, in Machordom et al. 2022**

Note: *T. sagamiensis* (Doflein, 1902) from Sagami Bay, Japan, is not included in the key. Considering the short description of the species and that the type is probably lost, the identity of this species remains questionable.

1. Less than 5 spines on branchial margin of carapace 2

— Five spines on branchial margin of carapace 48

2. Pleomere tergites unarmed 3

— Pleomere 2 tergite with pair of submedian spines or with additional spines along entire anterior ridge or restricted to lateral parts of ridge 20

3. Distinct carinae on lateral portion of sternites 6–7 4

— No carinae on sternites 6–7 7

4. Distomesial spine of antennal article 2 overreaching end of article 4. P1 fixed finger lacking spines on lateral margin, other than subterminal spines

*T. psylla* (Macpherson, 1994)

— Distomesial spine of antennal article 2 terminating at most in end of article 4. P1 fixed finger with row of spines on lateral margin 5

5. Distomesial spine of antennular article 1 shorter than distolateral. Three spines on branchial margin of carapace

*T. muscae* (Macpherson & de Saint Laurent 2002)

— Distal spines of antennular article 1 subequal. Four spines on branchial margin of carapace 6

6. Distomesial spine of the antennal article 2 barely reaching article 3. P1 movable finger with spines along mesial margin

*T. diritas* (Gallardo Salamanca & Macpherson, 2021)

— Distomesial spine of the antennal article 2 clearly exceeding article 3. P1 movable finger with only basal and distal spines on mesial margin

*T. vicina* (Komai, 2012)

7. Granules on lateral portion of sternite 7 8

— No granules on lateral portion of sternite 7 10

8. Distomesial spine of antennular article 1 slightly or moderately longer than distolateral spine. P1 movable finger with spines between basal and subterminal spines *T. kawamotoi* (Osawa & Okuno, 2002)

— Distomesial spine of antennular article 1 distinctly smaller than distolateral spine. P1 movable finger without spines between basal and subterminal spines 9

9. P1 fixed finger with row of spines along lateral margin. Movable finger with some proximal spines along mesial margin. *T. longiquus* (Komai, 2011)

— P1 fixed finger with distal spines only. Movable finger with proximal and distal spines on mesial margin  *T. hyalina* (Macpherson, 1994)

10. Distomesial spine of antennal article 1 very long, exceeding end of antennal peduncle *T. jurunjurun* (McCallum et al. 2021)

— Distomesial spine of antennal article 1 small or moderately long, not reaching end of antennal peduncle 11

11. Distal spines of antennular article 1 subequal or distomesial longer than distolateral 12

— Distomesial spine of antennular article 1 shorter than distolateral spine 18

12. Antennular article 1 long and slender, clearly exceeding eyes

***T. diluta* sp. nov.**

— Antennular article 1 barely reaching end of eyes 13

13. Lateral branchial spines minute, and only first spine prominent. P1 fixed finger with numerous strong spines along lateral margin

*T. erugata* (Macpherson, 2006)

— Lateral branchial margin with 4 well developed spines. P1 fixed finger unarmed or with small spines along lateral margin 14

14. Front margin oblique 15

— Front margin transverse 17

15. Distomesial spine of antennal article 1 exceeding article 3. Dorsal surface of P1 palm unarmed *T. fornacis* (Macpherson, 2006)

— Distomesial spine of antennal article 1 not exceeding article 3. Dorsal surface of P1 palm with numerous spines 16

16. P2 long and slender; merus 6 times as long as broad; dactylus 5 times as long as broad. One branchial dorsal spine on each side

*T. descensa* (Macpherson, 2006)

— P2 moderately long; merus 4 times as long as broad; dactylus 4 times as long as broad. Two branchial dorsal spines on each side ***T. brevitas* sp. nov**.

17. Supraocular spines well-developed, reaching or overreaching midlength of eyes *T. sentai* (Baba, 1986)

— Supraocular spines small, clearly not reaching midlength of eyes

*T. minuta* (Macpherson, 1993)

18. Rostrum dorsally not carinate *T. paucistria* (Komai, 2012)

— Rostrum dorsally carinate 19

19. Dorsal surface of carapace with anterobranquial and postcervical spines

*T. trigonocornus* (Komai, 2012)

— Dorsal surface of carapace without anterobranquial and postcervical spines

***T. microtes* sp. nov.**

20. Carinae on posterolateral part of sternal plastron 21

— No carinae on posterolateral part of sternal plastron 32

21. Carinae on lateral part of sternite 7 only *T. aulakodes* (Macpherson, 2006)

— Carinae at least on lateral parts of sternites 6-7 22

22. Distomesial and distolateral spines of antennular article 1 subequal 23

— Distomesial spine of antennular article 1 much smaller than distolateral spine 25

23. Antennular article 1, excluding distal spines, clearly exceeding eyes

*T. lenticularis* (Macpherson & de Saint Laurent, 1991)

— Antennular article 1, excluding distal spines, not exceeding eyes 24

24. Long lateral spine of antennular article 1 exceeding distal spines. Rostrum 0.4 carapace length *T. maculata* (Komai, 2012)

— Long lateral spine of antennular article 1 not exceeding distal spines. Rostrum 0.5 carapace length *T. ignea*  (Macpherson, 2006)

25. Carapace branchial margin with 4 spines. 26

— Carapace lateral margin with 3 spines. 29

26. Width of cornea about 1/4 distance between anterolateral spines of carapace *T. ocellata* (Macpherson & de Saint Laurent, 1991)

— Width of cornea more than 1/3 distance between anterolateral spines of carapace 27

27. Second spine of carapace lateral margin well developed, slightly shorter than first (anterolateral), subequal to third. Carinae on lateral parts of sternites 5–7

*T. pulchra* (Macpherson & de Saint Laurent, 1991)

— Second spine of carapace lateral margin much smaller than first (anterolateral) and third. Carinae on lateral parts of sternites 6–7 28

28. P1 carpus more than 3 times longer than wide

*T. longicheles* (Macpherson & de Saint Laurent, 1991)

— P1 carpus twice longer than wide *T. ommata* (Macpherson, 2004)

29. First anterolateral spine of carapace reaching level of sinus between rostral and supraocular spines. P2 propodus more than twice length of dactylus

*T. polynoe* (Macpherson & de Saint Laurent, 1991)

— First anterolateral spine of carapace falling short of level of sinus between rostral and supraocular spines. P2 propodus less than twice length of dactylus

30

30. Postcervical spine absent *T. cristulata* (Macpherson et al. 2017)

— Postcervical spine absent. 31

31. P1 fixed finger with some spines along proximal half of lateral margin. merus 0.5 length of that of P2, usually unarmed dorsally

*T. kapala* (Ahyong & Poore, 2004)

— P1 fixed finger with proximal half of lateral margin unarmed. P4 merus 0.9 length of that of P2, usually with row of dorsal spines.

*T. rufiantennulata* (Baba, 1969)

32. Pleomere 2 tergite with spines restricted to lateral parts of anterior ridge

*T. longinquus* (Komai, 2011)

— Pleomere 2 tergite with spines distributed along entire anterior ridge or with 2 median spines only 33

33. Granules on posterolateral part of sternal plastron 34

— No granules on posterolateral part of sternal plastron 42

34. Granules on posterolateral part of sternites 6-7 35

— Granules on posterolateral part of sternite 7 37

35. Antennular article 1, excluding distal spines, reaching end of cornea

*T. volantis* (Macpherson, 2004)

— Antennular article 1, excluding distal spines, clearly exceeding end of cornea

36

36. Width of cornea 1/4 distance between anterolateral spines of carapace

*T. rogeri* (Macpherson, 1994)

— Width of cornea half distance between anterolateral spines of carapace

*T. pasithea* (Macpherson & de Saint Laurent, 1991)

37. Distomesial spine of antennular article 1 slightly or distinctly longer than distolateral spine 38

— Distomesial spine of antennular article 1 shorter than or subequal to distolateral spine 40

38. Dorsal surface of carapace without anterobranquial and postcervical spines. Sternite 3 anteriorly produced, subtriangular with median sinus, breadth-length ratio 2.4 *T. evarne* (Macpherson & de Saint Laurent, 1991)

— Dorsal surface of carapace with anterobranquial and postcervical spines. Sternite 3 with anterior margin somewhat convex or feebly sinuous, breadth-length ratio 3.8 39

39. Mxp 3 merus with 2 subequal spines on flexor margin. P1 fingers about as long as or slightly shorter than palm *T. barbeti* (Galil, 1999)

— Mxp 3 merus with distal one of flexor marginal spines much smaller than proximal spine. P1 fingers distinctly longer than palm

*T. leptosyne* (Macpherson, 1994)

40. P1 movable finger with row of spines along whole margin

*T. tetracantha* (Macpherson et al. 2020)

— P1 movable finger with row of spines on proximal half of lateral margin 41

41. Antennular article 1, excluding distal spines, elongate, clearly exceeding end of cornea. Dorsal carapace surface without anterobranquial spines. Sternites 3 and 4 narrowly separated.  *T. gordoae* (Macpherson, 1994)

— Antennular article 1, excluding distal spines, moderately elongate, slightly exceeding end of cornea. Dorsal carapace surface with anterobranquial spines. Sternites 3 and 4 widely separated *T. consobrina* (Komai, 2012)

42. Distomesial spine of antennal article 2 falling short of end of article 4 43

— Distomesial spine of antennal article 2 overreaching article 4 45

43. Pleomere 2 tergite with spines along anterior ridge. Distomesial spine of antennal article 2 slightly overreaching end of article 3.

*T. rona* (Macpherson, 2013)

— Pleomere 2 tergite with 2 median spines on anterior ridge. Distomesial spine of antennal article 2 short, not reaching end of article 3. 44

44. Mxp 3 merus with with distal spine on extensor margin. P1 fingers clearly shorter than hand *T. psamathe* (Macpherson, 1994)

— Mxp 3 merus unarmed on extensor margin. P1 fingers as long as hand

*T. antliae* (Macpherson, 2006)

45. P2–4 dactyli unarmed at least on distal 1/3 of flexor margin 46

— P2–4 dactyli with movable spines along whole length of flexor margin 47

46. Pleomere 2 tergite with 4 pairs of spines. Mxp 3 merus unarmed on extensor distal margin. P1 fixed finger with 1 proximal and 1 subterminal spine only

*T. albiapicula* (Baba & Yu, 1987)

— Pleomere 2 tergite with 3 pairs of spines. Mxp 3 merus with spine on extensor distal margin. P1 fixed finger with a few spines between proximal and subterminal spines *T. masi* (Macpherson, 1994)

47. Distomesial spine of antennular article 1 distinctly shorter than distolateral spine. P2 merus with several spines on distal half of ventral margin

*T. nesiotes* (Macpherson, 1999)

— Distomesial and distolateral spines of antennular article 1 subequal. P2 merus with terminal spine only on ventral margin *T. zebra* (Macpherson, 1994)

48. Pleomere 2 tergite unarmed or with spines restricted to lateral parts of anterior ridge 49

— Pleomere 2 tergite with pair of submedian spines or additional spines distributed along anterior ridge 116

49. Granules on lateral portions of sternite 7 50

— No granules on sternal plastron 51

50. Supraocular spines clearly not reaching end of eyes. P2 dactylus length more than half length of propudus *T. redacta* (Ahyong, 2007)

— Supraocular spines nearly reaching end of eyes. P2 dactylus length at most half length of propudus *T. stigmatica* (Macpherson, 1994)

51. Pleomere 2 tergite with spines restricted to lateral sides 52

— Pleomere 2 tergite unarmed 60

52. Distomesial spine of antennal article 1 very long, reaching or nearly reaching end of antennule article 1 53

— Distomesial spine of antennal article 1 short or moderately long, clearly not reaching end of antennule article 1 56

53. Dorsal carapace surface without postcervical spine

*T. acantha* (Macpherson, 1994)

— Dorsal carapace surface with postcervical spine 54

54. Thoracic sternites 4-5 squamate. P1 carpus more than 4 times as long as broad *T. hastata* (Macpherson et al. 2021)

— Thoracic sternites 4-5 smooth. P1 carpus twice longer than broad 55

55. Distomesial spine of the antennular article 1 longer than distolateral

*T. jurunjurum* (McCallum et al. 2021)

— Distomesial and distolateral spines of the antennular article 1 subequal

*T. canopus* (Macpherson et al. 2020)

56. P2-4 dactyli unarmed at least on distal 1/3 of flexor margin 57

— P2-4 dactyli with movable spines along nearly entire length of flexor margin 58

57. Dorsal carapace surface without hepatic spines *T. japonica* (Stimpson, 1858)

— Dorsal carapace surface with hepatic spines *T. aequispina* Tiwari et al. 2023

58. Eyes small. Mxp3 merus unarmed on extensor margin

*T. abelloi* (Macpherson, 1994)

— Eyes large. Mxp3 merus with distal spine on extensor margin 59

59. Dorsal carapace surface with postcervical spine

*T. simulatrix* (Macpherson & Machordom, 2005)

— Dorsal carapace surface without postcervical spine

*T. spilota* (Macpherson, 1994)

60. Carapace with epigastric spines only on dorsal surface. 61

— Carapace with extra spines other than epigastric spines on dorsal surface 67

61. Distomesial spine of antennular article 1 shorter than distolateral 62

— Distomesial spine of antennular article 1 as long as or longer than distolateral

63

62. P2-4 dactyli short and unarmed at least on distal 1/3 of flexor margin. P2 dactylus 4.5 times longer than broad *T. stia* (Macpherson, 1994)

— P2-4 dactyli long and slender, with movable spines along nearly whole length of flexor margin. P2 dactylus 5.5 times longer than broad

*T. brachytes* (Macpherson, 1994)

63. P2-4 dactyli unarmed at least on distal 1/3 of flexor margin 64

— P2-4 dactyli with movable spines along whole length of flexor margin 66

64. Thoracic sternum squamate *T. vassiliyi* (Macpherson et al. 2021)

— Thoracic sternum smooth 65

65. Distomesial spine of antennal article 2 clearly exceeding antennal peduncle. Distomesial spine of antennal article 1 clearly exceeding antennal article 3

*T. runcinata* (Macpherson, 1994)

— Distomesial spine of antennal article 2 reaching end or not exceeding antennal peduncle. Distomesial spine of antennal article 1 not exceeding antennal article 3 *T. austrina* (Macpherson et al. 2017)

66. Mxp3 merus unarmed on extensor margin. P1 palm and fingers with strong spines; movable finger with spines along entire mesial margin

*T. apheles* (Macpherson, 2006)

— Mxp3 merus with distal spine on extensor margin. P1 palm and fingers with small spines; movable finger with proximal and distal spines only

*T. limatula* (Macpherson, 2004)

67. Distal spines of antennular article1 subequal or distomesial spine longer than distolateral 68

— Distomesial spine of antennular article1 shorter than distolateral 104

68. Distomesial spine of antennal article 2 reaching end or overreaching article 4

69

— Distomesial spine of antennal article 2 not overreaching end of article 4 92

69. Mxp 3 merus unarmed on extensor margin 70

— Mxp 3 merus with spine on extensor distal margin 75

70. Dorsal carapace surface with anterobranquial and postcervical spines. Eyes small; width of cornea 1/3 distance between anterolateral spines of carapace

*T. pseliophora* (Macpherson, 1994)

— Dorsal carapace surface without anterobranquial and/or postcervical spines. Eyes large; width of cornea slightly less than half distance between anterolateral spines of carapace 71

71. Dorsal carapace surface with postcervical spines

*T. leagora* (Macpherson, 1994)

— Dorsal carapace surface without postcervical spines 72

72. P2-4 dactyli unarmed at least on distal 1/3 of flexor margin 73

— P2-4 dactyli with movable spines along whole length of flexor margin 74

73. Dorsal carapace surface with anterobranquial spines

*T. disiunctus* (Komai, 20g11)

— Dorsal carapace surface without anterobranquial spines *T. alia* (Baba, 1994)

74. Supraocular spines exceeding eyes. First anterolateral spine of carapace exceeding sinus between rostral and supraocular spines

*T. moliae* (Macpherson, 1994)

— Supraocular spines not exceeding eyes. First anterolateral spine of carapace not exceeding sinus between rostral and supraocular spines

*T. atarapa* (Macpherson, 2013)

75. Front margin strongly oblique 76

— Front margin transverse or slightly oblique 78

76. Distal spines of antennular article1 subequal *T. clinata* (Macpherson, 1994)

— Distomesial spine of antennular article1 longer than distolateral 77

77. Pleomeres 2 and 3 tergites each with 2 transverse ridges in addition to anterior ridge. Dorsal carapace surface without protogastric spines behind epigastric spines *T. llenasi* (Macpherson, 2006)

— Pleomeres 2 and 3 tergites each with 1 transverse ridge in addition to anterior ridge. Dorsal carapace surface with some protogastric spines behind epigastric spines *T. pauxilla* (Macpherson, 2009)

78. P2–4 dactyli with spines along entire length (or ca. ¼) of flexor margin 79

— P2–4 dactyli unarmed at least on distal 1/3 of flexor margin 85

79. Dorsal carapace surface without anteriorbranchial spines 80

— Dorsal carapace surface with anteriorbranchial spines 81

80. Distomesial spine of antennular article 1 longer than distolateral. Distomesial spine of antennal article 2 reaching or slightly exceeding peduncle. First anterolateral spine of carapace not reaching sinus between rostral and supraocular spines *T. acola* (Macpherson, 2009)

— Distal spines of antennular article 1 subequal. Distomesial spine of antennal article 2 exceeding exceeding peduncle. First anterolateral spine of carapace reaching sinus between rostral and supraocular spines

*T. megalophthalma* Komai, 2011

81. Antennular article 1 long and slender, exceeding eyes ***T. macilenta* sp. nov.**

— Antennular article 1 only reaching end of eyes 82

82. Pleomeres 2-4 tergites each with 5-6 transverse ridges posterior to anterior ridge. 83

— Pleomeres 2-4 tergites each with 2-3 transverse ridges posterior to anterior ridge

84

83. P1 movable finger with spines along entire mesial margin. P2-4 moderately slender, less than 5 times longer than broad *T. multilineata* (Komai,2012)

— P1 movable finger with basal and distal spines only. P2-4 slender, more than 5 times longer than broad *T. mesembria* (Macpherson et al. 2017)

84. Distomesial spine antennal article 1 reaching end of article 2. Mxp3 merus with 2 spines along flexor margin.  *T. pavonis* (Macpherson, 2004)

— Distomesial spine antennal article 1 clearly exceeding end of article 2. Mxp3 merus with 3 spines along flexor margin

*T. pectinata* (Macpherson & Machordom, 2005)

85. Sternites 5–6 smooth, devoid of striae on surface 86

— Sternites 5–6 with striae 88

86. Dorsal carapace surface with postcervical spines. Median part of sternite 3 contiguous to sternite 4. Movable finger without spines between basal and subterminal spines *T. laevis* (Macpherson & Baba, 1993)

— Dorsal carapace surface without postcervical spines. Whole posterior margin of sternite 3 contiguous to sternite 4. Movable finger with spines between basal and subterminal spines 87

87*.* Pleomeres 2-4 tergites with 4-6 transverse ridges posterior to anterior ridge. Distomesial spine of antennular article 1 longer than distolateral. Distomesial spine antennal article 1 clearly exceeding end of article 2

*T. notata* (Macpherson, 1994)

— Pleomeres 2-4 tergites with 2-3 transverse ridges posterior to anterior ridge. Distal spines of antennular article 1 subequal. Distomesial spine antennal article 1 reaching or slightly exceeding end of article 2

*T. galaxaura* (Macpherson, 1996)

88. Distal spines of antennular article 1 subequal 89

— Distomesial spine of antennular article 1 longer than distolateral 90

89. Sternite 3 with anterior margin weakly bilobed, posterior margin broader than anterior margin of sternite 4 *T. caesura* (Macpherson & Baba, 1993)

— Sternite 3 with anterior margin produced into 2 distinct lobes, posterior margin narrower than anterior margin of sternite 4 91

90. Merus of Mxp3 with 3 spines along flexor margin. Mesial margin of movable finger of P1 with proximal and distal spines only. Supraocular spines not reaching end of eyes *T. messembria* (Macpherson et al. 2017)

— Merus of Mxp3 with 2 spines along flexor margin. Mesial margin of movable finger of P1 with spines along entire border. Supraocular spines reaching end of eyes *T. aurantiaca* (Macpherson et al. 2020)

91. Thoracic sternites 6-7 with few striae. Distomesial spine of antennal article 1 fully reaching end of article 3; distomesial spine of article 2 overreaching antennal peduncle by twice length of article 4 *T. sao* (Macpherson, 1994)

— Thoracic sternites 6-7 with numerous striae. Distomesial spine of antennal article 1 not reaching end of article 3; distomesial spine of article 2 overreaching antennal peduncle by length of article 4

*T. pherusa* (Macpherson & Baba, 1993)

92. P2–4 dactyli unarmed at least on distal 1/3 of flexor margin 93

— P2–4 dactyli with spines along entire length (or ca. ¼) of flexor margin 94

93. Thoracic sternite 3 narrower than anterior margin of sternite 4

*T. leeuwin* (McCallum et al. 2021)

— Thoracic sternite 3 wider than anterior margin of sternite 4

*T. samudrika* Tiwari et al. 2022

94. Dorsal carapace surface without anteriorbranquial spines 95

— Dorsal carapace surface with anteriorbranquial spines 97

95. Dorsal carapace surface without postcervical spines

*T. olivarae* (Macpherson, 1994)

— Dorsal carapace surface with postcervical spines 96

96. Front margin oblique. Distal spines of antennular article 1 subequal

*T. pusiola* (Macpherson, 1993)

— Front margin transverse. Distomesial spine of antennular article 1 slightly longer than distolateral spine *T. glabella* (Macpherson, 2000)

97. P1 fingers equal or shorter than palm 98

— P1 fingers distinctly longer than palm 102

98. Pleomeres 2-4 tergites with 0-1 transverse ridge posterior to anterior ridge. Distomesial spine of antennular article 1 longer than distolateral

*T. foresti* (Macpherson & de Saint Laurent, 1991)

— Pleomeres 2-4 tergites with 4-5 transverse ridges posterior to anterior ridge. Distal spines of antennular article 1 subequal 99

99. Sternite 3 with anterior margin nearly transverse bearing median notch

*T. arabica* (Tirmizi & Javed, 1992)

— Sternite 3 with anterior margin produced into 2 lobes 100

100. Front margin oblique 101

— Front margin transverse or slightly oblique *T. roshanei* (Tirmizi, 1966)

101. Antennal article 2 without spine on mesial margin. Carapace lateral margins feebly convex *T. munin* (Komai, 2011)

— Antennal article 2 with spine on mesial margin. Carapace lateral margins moderately convex  *T. bharuchai* Tiwari et al. 2023

102. Pleomeres 2-4 tergites with 0-1 transverse ridge posterior to anterior ridge

*T. mica* (Macpherson, 2009)

— Pleomeres 2-4 tergites with 3-5 transverse ridges posterior to anterior ridge. 103

103. P1 movable finger with row of spines along mesial margin. Distomesial spine of antennular article 1 longer than distolateral *T.* *osawai* (Komai, 2012)

— P1 movable finger with basal and distal spines only along mesial margin. Distal spines of antennular article 1 subequal *T. janetae* (Tirmizi & Javed, 1992)

104. Rostral spine more than distance between mid-cervical groove and sinus formed by rostral and supraocular spines *T. barangei* (Macpherson, 1994)

— Rostral spine distinctly shorter than distance between mid-cervical groove and sinus formed by rostral and supraocular spines 105

105. Mxp 3 merus unarmed on extensor margin 106

— Mxp 3 merus with distinct spine (sometimes obsolescent, e.g. *T. pulex*) on extensor distal margin 109

106. P1 carpus 5 times as long as broad. P2 more than 2.5 times length of carapace *T. offella* (Macpherson, 1996)

— P1 carpus about twice as long as broad. P2 less than twice length of carapace

107

107. Dorsal carapace surface with anteriorbranquial spines

*T. solitaria* (Komai, 2012)

— Dorsal carapace surface without anteriorbranquial spines 108

108. P2–4 dactyli unarmed at least on distal 1/3 of flexor margin. P1 carpus and palm slender *T. goga* (Macpherson et al. 2020)

— P2–4 dactyli with spines along entire length (or ca. ¼) of flexor margin. P1 carpus and palm stout *T. micula* (Macpherson, 1996)

109. P2–4 dactyli with movable spines along entire length of flexor margin. 110

— P2–4 dactyli unarmed at least on distal 1/4 of flexor margin 113

110. P1 fixed finger with well developed spines along lateral margin 111

— P1 fixed finger unarmed or with minute spines along lateral margin 112

111. P2-4 propodi 5.3-5.7 times longer than broad. Dactyli 5 times longer than broad ***T. pulex* sp. nov.**

— P2-4 propodi 4.0-4.6 times longer than broad. Dactyli 3.7-4.5 times longer than broad *T. leptitis* (Macpherson, 1994)

112. Rostrum dorsally carinate. Dorsal carapace surface with anteriorbranquial spines. Supraocular spines well developed, reaching or exceeding midlength of eyes. *T. alonsoi* (Macpherson, 1994)

— Rostrum dorsally convex. Dorsal carapace surface without anteriorbranquial spines. Supraocular spines very small, not reaching midlength of eyes

*T. pumila* (Macpherson, 2004)

113. P2 more than 2.5 times carapace length. P1 carpus more than 3 times longer than broad *T. proto* (Macpherson, 1994)

— P2 1.5-2.0 times carapace length. P1 carpus less than 3 times longer than broad 114

114. Rostrum triangular ……..*T. antonbruuni* (Tirmizi & Javed, 1980)

— Rostrum spiniform 115

115. Distomesial spine of antennal article 2 exceeding article 4. Dorsal carapace surface with anteriorbranquial spines. Rostrum dorsally convex .

*T. apodis* (Macpherson, 2004)

— Distomesial spine of antennal article 2 reaching end of article 3. Dorsal carapace surface without anteriorbranquial spines. Rostrum dorsally carinate

*T. parvula* (Macpherson, 1993)

116. Granules on posterolateral part of sternal plastron 117

— No granules on posterolateral part of sternal plastron 132

117. Granules on sternites 6–7 118

— Granules on sternite 7 only 122

118. Lateral parts of thoracic sternites covered with many small granules 119

— Lateral parts of thoracic sternites with a few coarse granules 121

119. Extensor margin of Mxp3 merus with distal spine. Antennular peduncle not exceeding cornea *T. taenia* (Macpherson, 1994)

— Extensor margin of Mxp3 merus unarmed. Antennular peduncle distinctly exceeding cornea 120

120. Granules on thoracic sternites 6 and 7 forming lines. Distal spines on antennular article 1 subequal *T. lineola* (Macpherson, 1994)

— Granules on thoracic sternites 6 and 7 homogeneously scattered. Distomesial spine on antennular article 1 longer than distolateral

*T. pontoporea* (Macpherson, 1994)

121. Distomesial spine on antennular article 1 slightly longer than distolateral. One basal and one distal spine on mesial margin of P1 movable finger

*T. idyia* (Macpherson, 1994)

— Distal spines on antennular article 1 subequal. Several spines along proximal half of mesial margin of P1 movable finger *T. tyche* (Macpherson, 1994)

122. Pleomere 3 tergite with 3-4 spines on anterior ridge

*T. macphersoni* (Cabezas et al. 2011)

— Pleomere 3 tergite unarmed 123

123. Distomesial spine on antennular article 1 shorter than distolateral

*T. armilla* (Macpherson, 1994)

— Distal spines on antennular article 1 subequal or distomesial spine slightly longer than distolateral 124

124. Dorsal carapace surface without anteriorbranquial spines 125

— Dorsal carapace surface with anteriorbranquial spines 126

125. Thoracic sternites 4-5 with numerous striae

*T. euripa* (Macpherson et al. 2017)

— Thoracic sternites 4-5 with a few striae or smooth

*T. heteracantha* (Ortmann, 1892)

126. P2–4 dactyli unarmed at least on distal 1/3 of flexor margin

*T. sirius* (Macpherson et al. 2020)

— P2–4 dactyli with movable spines along nearly entire length of flexor margin

127

127. Mxp 3 merus with spine on extensor distal margin 128

— Mxp 3 merus unarmed on extensor distal margin 131

128. Distomesial spine of antennal article 2 not overreaching antennal peduncle

*T. limula* (Macpherson & Baba, 1993)

— Distomesial spine of antennal article 2 overreaching antennal peduncle . 129

129. Sternite 4 with a few striae, sternites 5–6 smooth

*T. spinicruris* (Ahyong & Poore, 2004)

— Sternites 4–7 with arcuate striae 130

130. Coarse granules on lateral parts of sternite 7 *T. guttata* (Macpherson, 1994)

— Fine granules on lateral part of sternite 7 *T. honshuensis* (Benedict, 1902)

131. P2 dactylus 0.7 times as long as propodus. Front margin oblique

*T. squamifera* (Komai, 2012)

— P2 dactylus 0.5 times as long as propodus. Front margin transverse

*T. distiza* (Macpherson, 1994)

132. Pleomere 4 tergite with spines on anterior ridge 133

— Pleomere 4 tergite unarmed 134

133. P1 carpus about 4-5 times as long as broad *T. gilii* (Macpherson, 1993)

— P1 carpus about 3 times as long as broad *T. babai* (Tirmizi & Javed, 1976)

134. Pleomere 3 tergite with spines on anterior ridge. 135

— Pleomere 3 tergite unarmed 138

135. Dorsal carapace surface with anteriorbranquial spines

*T. armata* (Baba, 1988)

— Dorsal carapace surface without anteriorbranquial spines 136

136. Distomesial spine of antennal article 2 exceeding antennal peduncle

*T. prominula* (Baba, 1988)

— Distomesial spine of antennal article 2 not exceeding antennal peduncle 137

137. Distomesial spine on antennular article 1 shorter than distolateral

*T. jubata* (Macpherson, 2009)

— Distal spines on antennular article 1 subequal *T. spinulifera* (Miers, 1884)

138. Pleomere 2 tergite with pair of submedian spines only on anterior ridge 139

— Pleomere 2 tergite with 6 or more spines along enrire anterior ridge 142

139. Distal spines on antennular article 1 subequal

*T. philippinensis* (Macpherson & Baba, 1993)

— Distomesial spine on antennular article 1 shorter than distolateral 140

140. Rostrum dorsally carinate *T. stomifera* (Macpherson et al. 2017)

— Rostrum dorsally not carinate 141

141. Dorsal carapace surface armed only with epigastric spines

*T. micra* (Macpherson et al. 2017)

— Dorsal carapace surface armed with other spines, in addition to epigastric spines *T. antliae* (Macpherson, 2006)

142. Distomesial and distolateral spines of antennular article 1 subequal in size, or distomesial longer than distolateral 143

— Distomesial spine of antennular article 1 shorter than distolateral 155

143. Distomesial spine of antennal article 1 overreaching article 3 144

— Distomesial spine of antennal article 1 not overreaching article 3 148

144. Mxp 3 merus with spine on extensor distal margin. Movable finger with spines between basal and subterminal spines on mesial margin 145

— Mxp 3 merus unarmed on extensor distal margin. Movable finger without spines between basal and subterminal spines 146

145. Sternal plastron with numerous arcuate striae. P2–4 dactyli relatively broad (length-breadth ratio ca. 4.2) *T. eudora* (Macpherson & Baba, 1993)

— Sternal plastron less strigose, no striae on sternite 6. P2–4 dactyli slender (length-breadth ratio ca. 6.5) *T. melite* (Macpherson & Baba, 1993)

146. P2 dactylus unarmed on distal 1/4 of flexor margin. Dorsal carapace surface without anteriorbranquial spines *T. oritea* (Macpherson & Baba, 1993)

— P2 dactylus unarmed on nearly distal half of flexor margin. Dorsal carapace surface with anteriorbranquial spines 147

147. Suprocular spines exceeding eyes. Distomesial spine of antennal article 1 exceeding antennal peduncle *T. strigosa* (Macpherson et al. 2020)

— Suprocular spines not exceeding eyes. Distomesial spine of antennal article 1 not exceeding antennal peduncle *T. striola* (Macpherson & Baba, 1993)

148. Mxp 3 merus with distinct spine on extensor distal margin 149

— Mxp 3 merus unarmed on extensor distal margin 152

149. Distomesial and distolateral spines of antennular article 1 subequal in size

150

— Distomesial spine of antennular article 1 longer than distolateral 151

150. Thoracic sternites with numerous striae

*T. nesaea* (Macpherson & Baba, 1993)

— Thoracic sternites with a few striae *T. sphinx* (Macpherson & Baba, 1993)

151. Sternite 4 contiguous to median part of posterior margin of sternite 3. P2–4 dactyli without corneous spine at base of terminal claw

*T. agave* (Macpherson & Baba, 1993)

— Sternite 4 contiguous to most part of posterior margin of sternite 4. P2–4 dactyli with corneous spine at base of terminal claw *T. latior* (Baba, 2005)

152. P2 dactylus unarmed at least on distal 1/3 of flexor margin 153

— P2 dactylus with movable spines nearly along entire flexor margin 154

153. Dorsal carapace surface armed only with epigastric spines

*T. carinata* (Baba, 2005)

— Dorsal carapace surface armed with other spines, in addition to epigastric spines *T. semoni* (Ortmann, 1894)

154. Dorsal carapace surface without anterobranquial and postcervical spines

*T. collier* (Ahyong, 2007)

— Dorsal carapace surface with anterobranquial and postcervical spines

*T. thoe* (Macpherson, 1994)

155. Tubercle-like small spines on anterior branchial region. Antennal peduncle reduced in size *T. tuberculata* (Henderson, 1885)

— No spine or at most 1 or 2 spines on anterior branchial region. Antennal peduncle well developed 156

156. Distomesial spine of antennal article 2 never reaching end of article 4

157

— Distomesial spine of antennal article 2 reaching or overreaching end of article 4 164

157. P2 dactylus unarmed at least on distal 1/3 of flexor margin 158

— P2 dactylus with movable spines along entire length of flexor margin 160

158. Antennular peduncle ending at level of or slightly exceeding corneae. Dorsal carapace surface without postcervical spines *T. caeli* (Cabezas et al. 2009)

— Antennular peduncle clearly exceeding corneae. Dorsal carapace surface armed with postcervical spines 159

159. P2 three times carapace length. P1 4.5 times carapace length

*T. lailai* (Cabezas et al. 2009)

— P2 twice carapace length. P1 2.5 times carapace length

*T. parca* (Macpherson, 1996)

160. Supraocular spines long reaching end of eyes 161

— Supraocular spines short not reaching midlength of eyes 163

161. P2 dactylus short, 3 times as long as broad, *T. insularis* (Macpherson, 1999)

— P2 dactylus long and slender, more than 4 times as long as broad 162

162. Dorsal carapace surface with numerous ridges, e.g. branchial margins with ca. 10 ridges *T. koyo* (Komai, 2011)

— Dorsal carapace surface with a few ridges, e.g. branchial margins with ca. 5 ridges *T. rupicola* (Lin & Chan, 2005)

163. P1 palm more than 2.5 times as long as broad

*T. fasciata* (Macpherson, 2006)

— P1 palm at most 1.7 times as long as broad

*T. parile* (Macpherson & Machordom, 2005)

164. Supraocular spines reaching or nearly reaching end of eyes. Dorsal carapace surface armed with anterobranchial spines 165

— Supraocular spines clearly not reaching end of eyes. Dorsal carapace surface without anterobranchial spines 166

165. P1 movable finger with spines along mesial margin. P1 carpus twice longer than broad *T. dispar* (Macpherson & Baba, 1993)

— P1 movable finger unarmed, except proximal and distal spines along mesial margin. P1 carpus 4 times as long as broad *T. hydri* (Macpherson et al. 2020)

166. Distomesial spine of antennal article 2 clearly exceeding peduncle. P2-4 dactyli 5.5 times longer than high *T. squarrosa* (Macpherson, 2009)

— Distomesial spine of antennal article 2 reaching end of peduncle. P2-4 dactyli 7.5 times longer than high *T. mendagnai* (Cabezas et al. 2009)

**Key to species of the genus *Typhlonida* Macpherson & Baba, in Machordom et al. 2022**

1. Granules on posterolateral part of sternal plastron 2

— No granules on posterolateral part of sternal plastron 5

2. Pleomere 3 tergite with spines on anterior ridge 3

— Pleomere 3 tergite unarmed 4

3. Granules on sternites 6–7. Pleomere 4 tergite with 2 median spines on anterior ridge *T. guineae* (Miyake & Baba, 1970)

— Granules on sternite 7 only. Pleomere 4 tergite unarmed

*T. valida* (Smith, 1883)

4. Movable finger of P1 with spines along entire mesial margin. First anterolateral spine of carapace short not reaching sinus between rostral and supraocular spines *T. propinqua* (Faxon, 1893)

— Movable finger of P1 with proximal and distal spines on mesial margin. First anterolateral spine of carapace long reaching sinus between rostral and supraocular spines *T. sanctipauli* (Henderson, 1885)

5. Pleomere 2 tergite with spines along anterior ridge. 6

— Pleomere 2 tergite unarmed along anterior ridge. 18

6. Pleomere 3 tergite with spines along anterior ridge 7

— Pleomere 3 tergite unarmed along anterior ridge 8

7. Pleomere 4 tergite with spines along anterior ridge

*T. constricta* (A. Milne Edwards, 1880)

— Pleomere 4 tergite unarmed along anterior ridge

*T. miles* (A. Milne Edwards, 1880)

8. Cornea clearly wider than ocular peduncle 9

— Cornea as wide as or narrower than ocular peduncle 10

9. First anterolateral spine of carapace long, reaching sinus between rostral and supraocular spines. Antennal article 1 with distomesial spine long, exceeding midlength of article 2 *T. lanciaria* (Cabezas et al. 2011)

— First anterolateral spine of carapace short, clearly not reaching sinus between rostral and supraocular spines. Antennal article 1 with distomesial spine short, not reaching midlength of article 2 *T. pygmaea* (Macpherson, 1996)

10. Dorsal carapace surface with one anterobranchial spine on each side 11

— Dorsal carapace surface without anterobranchial spine spines 13

11. Dorsal carapace surface with one minute postcervical spine on each side 12

— Dorsal carapace surface without postcervical spines

*T. parvioculata* (Baba, 1982)

12. Antennal article 3 with distomesial spine *T.* *victoria* (Melo-Filho, 1996)

— Antennal article 3 unarmed *M. magniantennulata* (Baba & Türkay, 1992)

13. P1 setose, densely covered with plumose setae *T. crassa* (Baba, 1982)

— P1 non setose, with a few plumose setae only 14

14. Antennal article 3 with distomesial spine 15

— Antennal article 3 unarmed 17

15. Dorsal surface of P1 palm unarmed *T. galalala* (McCallum et al. 2021)

- Dorsal surface of P1 palm with some spines 16

16. P1 fixed finger unarmed, with only small distal spines. ***T. eluminata* sp. nov.**

— P1 fixed finger with spines along entire lateral margin

*T. microphthalma* (A. Milne Edwards, 1880)

17. Supraocular spines short, clearly not reaching end of corneae ……….

*T. alaos* Macpherson et al. 2023

— Supraocular spines barely reaching or exceeding end of corneae 18

18. Distomesial spine of antennal article 1 reaching end of article 2. Supraocular spines barely reaching end of cornea *T. perlata* (Benedict, 1902)

— Distomesial spine of antennal article 1 not reaching end of article 2. Supraocular spines exceeding end of cornea *T. typhle* (Macpherson, 1994)

19. Antennal article 3 with distomesial spine *T. watatsumi* (Komai, 2014)

— Antennal article 3 unarmed 20

20. Dorsal carapace surface with one postcervical spine on each side

*T. devestiva* (Macpherson, 2006)

— Dorsal carapace surface without postcervical spines 21

21. Distomesial spine of antennal article 1 very short, clearly not reaching midlength of article 2 22

— Distomesial spine of antennal article 1 reaching or exceeding midlength of article 2 25

22. Antennular article 1 elongate *T. oblonga* (Macpherson, 2006)

— Antennular article 1 not elongate 23

23. First lateral spine of carapace mesial to anterolateral angle 24

— First lateral spine of carapace at anterolateral angle …………………………….. ………………………………. *T. ampliantennulata* (Komai, 2011)

24. P1 palm longer than fingers *T. tiresias* (Macpherson, 1994)

— P1 palm shorter than fingers *T. subcaeca* (Bouvier, 1922)

25. First lateral spine of carapace mesial to anterolateral angle. Merus of Mxp3 without strong median spine on flexor margin *T. milindi* Periasamy et al. 2023

— First lateral spine of carapace at anterolateral angle. Merus of Mxp3 with strong median spine on flexor margin *T. alba* (Liu et al. 2020)
